# BMP4 triggers regulatory circuits specifying the cardiac mesoderm lineage

**DOI:** 10.1242/dev.201450

**Published:** 2023-05-22

**Authors:** Pavel Tsaytler, Jinhua Liu, Gaby Blaess, Dennis Schifferl, Jesse V. Veenvliet, Lars Wittler, Bernd Timmermann, Bernhard G. Herrmann, Frederic Koch

**Affiliations:** ^1^ Department of Developmental Genetics, Max Planck Institute for Molecular Genetics, 14195 Berlin, Germany; ^2^ Sequencing Core Facility, Max Planck Institute for Molecular Genetics, 14195 Berlin, Germany

**Keywords:** Cardiac mesoderm, Bmp4, Differentiation, Heart, WNT signaling, Cardiac enhancers, Mouse

## Abstract

Cardiac lineage specification in the mouse is controlled by TGFβ and WNT signaling. From fly to fish, BMP has been identified as an indispensable heart inducer. A detailed analysis of the role of Bmp4 and its effectors Smad1/5, however, was still missing. We show that Bmp4 induces cardiac mesoderm formation in murine embryonic stem cells *in vitro*. Bmp4 first activates *Wnt3* and upregulates *Nodal*. pSmad1/5 and the WNT effector Tcf3 form a complex, and together with pSmad2/3 activate mesoderm enhancers and *Eomes*. They then cooperate with Eomes to consolidate the expression of many mesoderm factors, including *T*. Eomes and T form a positive- feedback loop and open additional enhancers regulating early mesoderm genes, including the transcription factor *Mesp1*, establishing the cardiac mesoderm lineage. In parallel, the neural fate is suppressed. Our data confirm the pivotal role of Bmp4 in cardiac mesoderm formation in the mouse. We describe in detail the consecutive and cooperative actions of three signaling pathways, BMP, WNT and Nodal, and their effector transcription factors, during cardiac mesoderm specification.

## Teaser

**Summary:** Bmp4 induces cardiac mesoderm formation in mESCs, whereby Bmp4 activates Nodal and Wnt3, and all three signaling pathways cooperate in activating mesodermal enhancers and cardiac progenitor genes.

## INTRODUCTION

In mammals, mesodermal cell lineages are derived from pluripotent epiblast cells that undergo epithelial-to-mesenchymal transition (EMT) in the primitive streak (PS) or tailbud, and ingress (Tam and Loebel, 2007). Cells originating from the proximal PS give rise to cardiac mesoderm progenitors, whereas the distal PS generates paraxial mesoderm (Lawson, 1999). Mesoderm formation is controlled by several inducers, in particular members of the TGFβ and WNT families, each activating its own signaling pathway and target transcription factors (TFs), primarily the essential regulators Eomes and T (brachyury) (Chu et al., 2004; Brennan et al., 2001; Russ et al., 2000; Arnold et al., 2008; Herrmann et al., 1990).

TGFβ signaling in the PS is activated by Bmp4 and Nodal, and their downstream effectors – Smad TFs (Winnier et al., 1995; Conlon et al., 1994; Gaarenstroom and Hill, 2014). Both *Nodal*- and *Smad2/3*- (the Nodal effectors) deficient embryos do not form mesoderm and fail to gastrulate (Conlon et al., 1994; Dunn et al., 2004). *Bmp4*-deficient embryos display variable phenotypes; most mutant embryos do not proceed beyond the egg cylinder stage and form little or no mesoderm (Winnier et al., 1995). Furthermore, embryos lacking the Bmp4 receptor *Bmpr1a* do not form mesoderm and die before E9.5, suggesting that BMP signaling is essential for gastrulation (Mishina et al., 1995). Bmp4 induces phosphorylation of partially redundant Smad1/5 proteins (Arnold et al., 2006). *Smad5* mutant embryos showed delayed cardiac development and abnormal heart looping, but neither *Smad1* nor *Smad5* knockout prevented mesoderm formation *in toto* (Tremblay et al., 2011; Chang et al., 2000). Similarly, a high proportion of *Smad1/5* double heterozygous mutant embryos displayed heart looping defects (Arnold et al., 2006). *Smad1/5* double homozygous mutant embryos have not been reported.

Smad1/5 and Smad2/3 interact with the common co-factor Smad4, allowing DNA binding and transcriptional regulation of target genes (Gaarenstroom and Hill, 2014; Massague et al., 2005). Embryos lacking *Smad4* do not form mesoderm and fail to gastrulate (Sirard et al., 1998). However, conditional depletion of *Smad4* in the early epiblast did not prevent cardiac lineage specification and formation of a rudimentary heart, as well as somite formation, whereas primordial germ cells or an allantois did not form (Chu et al., 2004). This data suggested that *Smad4* is dispensable for heart development in the mouse. In contrast, human *Smad4* mutant embryonic stem cells (ESCs) differentiated *in vitro* fail to form cardiac mesoderm or cardiomyocytes (Xu et al., 2019). Moreover, in other vertebrates such as *Xenopus*, zebrafish and chick, BMP has been shown to play an essential role in cardiac mesoderm induction and heart development as well (Shi et al., 2000; de Pater et al., 2012; Schultheiss et al., 1997). In *Drosophila*, the BMP homolog dpp is essential for heart induction too, pointing to a conserved role of BMP in heart development in evolution (Frasch, 1995). Therefore, it appears highly unlikely that BMP is dispensable for cardiac mesoderm formation in mouse.

TGFβ pathways crosstalk with other signaling cascades, and Smad TFs interact with various factors in a context-dependent manner (Chen et al., 2008; Morikawa et al., 2016). WNT signaling, activated concurrently with TGFβ in the PS, is essential for proper PS and mesoderm formation (Liu et al., 1999). WNT and TGFβ pathways have been shown to interact in various model organisms (Reid et al., 2012; Trompouki et al., 2011). In differentiating ESCs, Smad2/3 and TCF factors co-occupy mesendodermal gene enhancers, suggesting cooperation between WNT and SMAD pathways in mesendoderm (Wang et al., 2017; Funa et al., 2015).

Early mesoderm formation is orchestrated by the T-box family members Eomes and T (Costello et al., 2011; Papaioannou, 2014). Although T is a pan-mesodermal TF highly expressed in the PS and posterior mesoderm, it is not essential for cardiac mesoderm formation, as embryos lacking *T* develop beating heart structures (Concepcion and Papaioannou, 2014). Embryos with epiblast-specific *Eomes* deletion, on the other hand, fail to express cardiac marker genes, suggesting that *Eomes* is essential for specification of cardiac progenitors (Costello et al., 2011). In addition, *Eomes* and *T* are required for suppressing the neural differentiation program in nascent mesoderm (Tosic et al., 2019).

In this study, we have analyzed the process of Bmp4-induced early cardiac mesoderm specification in mouse (m) ESCs by functional characterization of the control mechanisms employed by the consecutively activated mesodermal key TFs Smad1/5, Smad2, Eomes and T.

## RESULTS

### Cardiac progenitor genes are co-bound by pSmad1/5 and pSmad2, and require Smad4 for expression

**Fig. 1.**
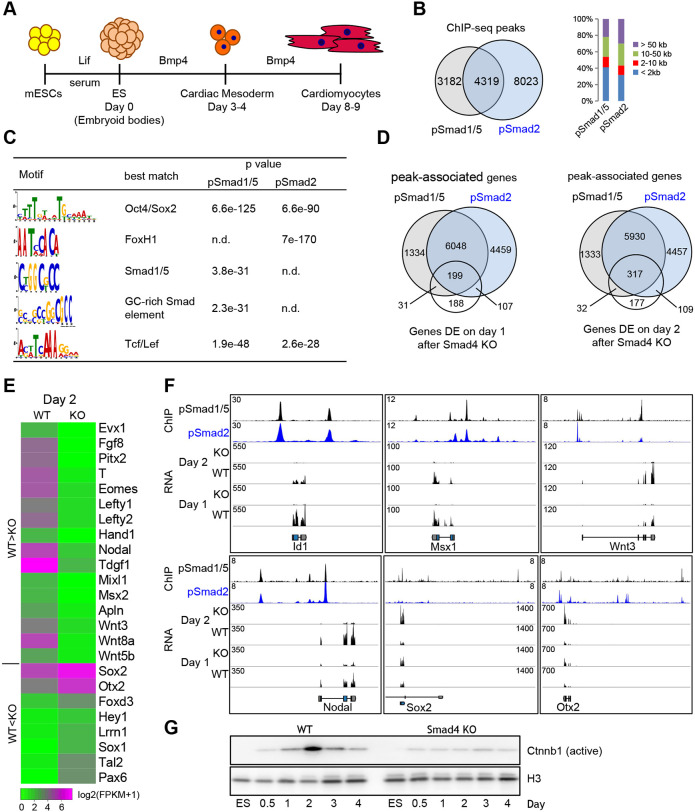
**Cardiac progenitor genes are co-bound by pSmad1/5 and pSmad2, and require Smad4 for expression.** (A) Schematic of the *in vitro* differentiation course of mESCs to cardiac mesoderm. ES refers to embryoid bodies stage. (B) Venn diagram showing overlap between pSmad1/5 and pSmad2 DNA binding sites in day 2 ME cells (left). Barplot shows the quantification of distances between binding sites and nearest gene promoters (right). (C) Top *de novo* motifs discovered at pSmad binding sites. n.d., not detected. (D) Venn diagram showing overlap between genes bound by pSmad1/5 and/or pSmad2, and genes differentially expressed between WT and *Smad4* KO cells at day 1 or day 2 of differentiation. (E) Heatmap representation of the expression of selected pSmad1/5/2 direct target genes in WT or *Smad4* KO cells at day 2 of differentiation (RNA-seq, average of two replicates). (F) Snapshots of ChIP-seq and RNA-seq tracks in WT or *Smad4* KO ME cells at selected genomic loci. See also Fig. S1 and Tables S1-S3. (G) Western blot analysis of time-dependent nuclear accumulation of active Ctnnb1 in WT and *Smad4* KO cells. Histone H3 (H3) served as loading control. See also Fig. S1 and Tables S1-3.

In the mouse embryo, cardiac mesoderm is formed from cells located in the posterior epiblast, which are exposed to Bmp4 secreted from the adjacent extra-embryonic ectoderm. However, in *in vitro* studies, cardiac mesoderm is commonly induced by activin A (ActA) and/or WNT signaling (Tosic et al., 2019; Zhao et al., 2019). We set out to generate cardiac mesoderm (ME) formation by exposing mESCs to Bmp4, thereby inducing processes occurring in the posterior PS (Fig. 1A; Fig. S1A,B). The dynamics of marker gene expression reflected mesoderm formation in the embryo. The pluripotency genes *Dppa3*, *Sox2* and *Esrrb* were downregulated after day 1 (Fig. S1C), followed by upregulation of nascent mesoderm control genes *Nodal*, *Eomes*, *Mixl1*, *Wnt3* and *T* on day 2 and 3. The latter was paralleled by upregulation of the EMT genes *Zeb2* and *Snai1*, as well as of the lateral mesoderm and cardiac progenitor genes *Mesp1*, *Foxf1*, *Kdr*, *Gata4*, *Hand1*, and *Isl1* on day 4 (Fig. S1C,D). Neuroectoderm (e.g. *Sox1*, *Pax6*), endoderm (*Sox17*, *Sox7*), trophoblast (*Cga*, *Gcm1*), allantois (*Cdx4*, *Tie1*) and hematopoietic (*Tal1*, *Runx1*) lineage genes were not or hardly expressed, confirming the specificity of the approach (Fig. S1E). On day 6, around two-thirds (65.3%) of the cells expressed the cardiomyocyte-specific gene *Tnnt2* (Fig. S1F) and, on day 8, all of the colonies were contracting and expressed markers of committed cardiomyocytes, Actc1 and Myl7, reflecting the high efficiency of the differentiation scheme (Fig. S1G; Movie 1). Thus, our *in vitro* system faithfully copied mesoderm induction, EMT and cardiac mesoderm formation in the mouse embryo.

We assessed Smad1/5 and Smad2/3 activation by monitoring their phosphorylation levels, which peaked on day 2 or day 3, respectively (Fig. S1H). To gain insight into the mechanisms of SMAD-mediated transcriptional regulation, we first detected DNA binding sites (peaks) of Smad TFs in wild-type (WT) cells on differentiation day 2 (ME, day 2) using ChIP-seq, yielding 7575 phospho-Smad1/5 (pSmad1/5) and 12,423 phospho-Smad2 (pSmad2) peaks (Fig. S1I). Upon phosphorylation, pSmad1/5 and pSmad2/3 form complexes with Smad4, essential for regulation of target genes (Massague et al., 2005). Therefore, depletion of *Smad4* is expected to reduce or eliminate DNA-bound pSmad1/5 and pSmad2 complexes. Indeed, using Bmp4-treated *Smad4* knockout (KO) cells, we detected only 493 pSmad1/5 and 554 pSmad2 peaks. These peaks showed low peak-calling confidence and were excluded from further analyses (Fig. S1I). We found that 4418 (58%) of the 7501 pSmad1/5 and 8425 (68%) of the 12,342 pSmad2 peaks were located outside of gene promoters, indicating putative enhancer regions (Fig. 1B). To substantiate this assumption we plotted the corresponding profiles of enhancer-associated chromatin marks detected in the posterior PS of the embryonic day (E)7 mouse embryo (Yang et al., 2019; Fig. S1J,K). A large fraction of the promoter-far binding sites of pSmad1/5 and pSmad2 were flanked by H3K27Ac and H3K4me1 marks, confirming that the majority of pSmad binding sites in ME cells are located in active enhancers. pSmad1/5 and pSmad2 peaks were enriched in previously reported GC-rich Smad1 or FoxH1 motifs, respectively, validating the specificity of the antibodies (Morikawa et al., 2011; Fig. 1C). Notably, both peak sets displayed strong enrichment of the Tcf/Lef motif associated with WNT signaling. The Oct4/Sox2 motif was significantly enriched as well, pointing to occupancy of these enhancers by pluripotency factors before differentiation.

Next, we compared the target genes of the two SMAD pathways and assessed their biological functions using Gene Ontology (GO) term analysis. Whereas genes bound by either pSmad1/5 or pSmad2 were only enriched in GO terms associated with nervous system development, genes co-bound by both pSmad1/5 and pSmad2 were also enriched in WNT signaling, mesoderm and heart development, and EMT terms (Fig. 1D; Table S1). This suggests that induction of the cardiac mesodermal program and EMT is mediated by the integration of both BMP and Nodal signaling.

We identified putative direct target genes of SMAD by intersecting pSmad1/5- and pSmad2-bound genes with genes differentially expressed (DE) between *Smad4* KO and WT ME cells. *Smad4* depletion disrupted the majority of pSmad1/5 and pSmad2 DNA binding (Fig. S1I), and neither Bmp4 nor ActA induced Eomes or T in KO cells (Fig. 1E; Fig. S1L). Therefore, in *in vitro* differentiated cells, loss of *Smad4* blocks both BMP and Nodal signaling, and thus allows the identification of direct target genes of both Smad1/5 and Smad2 (Fig. S1M).

Most putative direct targets were co-bound by pSmad1/5 and pSmad2 (Fig. 1D). Using GO term analysis, direct targets of pSmad1/5 and pSmad2 (Smad1/5/2) again were enriched in WNT signaling, mesoderm formation and heart development terms, whereas repressed targets were associated with nervous system development (Table S2). In contrast, direct target genes of either pSmad1/5 or pSmad2 alone did not show enrichment in any biological functions. Furthermore, the mesoderm control genes *Eomes*, *T*, *Tbx3*, *Fgf8*, *Mixl1*, *Evx1* and *Hand1*, and WNT family members *Wnt3*, *Wnt8a* and *Wnt5b* were among the most strongly activated Smad1/5/2 targets, and their expression was almost entirely abolished in *Smad4* KO cells. In contrast, the neural fate genes *Sox1*, *Sox2*, *Tal2* and *Pax6* were among the most strongly repressed Smad1/5/2 targets (Fig. 1E,F; Fig. S1M).

As *Wnt3* displayed transient upregulation during ME differentiation (Fig. S1C), and together with *Wnt8a* and *Wnt5b* was activated by Smads, we assessed whether all WNT signaling is abolished in the absence of SMAD signaling. We found that nuclear accumulation of active Ctnnb1 (β-catenin) peaked in day 2 WT ME cells and was strongly reduced in *Smad4* KO cells (Fig. 1G). Thus, in Bmp4-induced mesoderm WNT activation requires SMAD signaling.

The combined data demonstrate that during mesoderm formation *in vitro* Smad4 is essential for transcriptional activity of pSmad1/5 and pSmad2, and suggest a crucial role of pSmad1/5/2 cooperation in the activation of early cardiac progenitor and repression of neural genes.

### Cardiac progenitor and cardiomyocyte formation require pSmad1/5, pSmad2/3, and WNT signaling

**Fig. 2.**
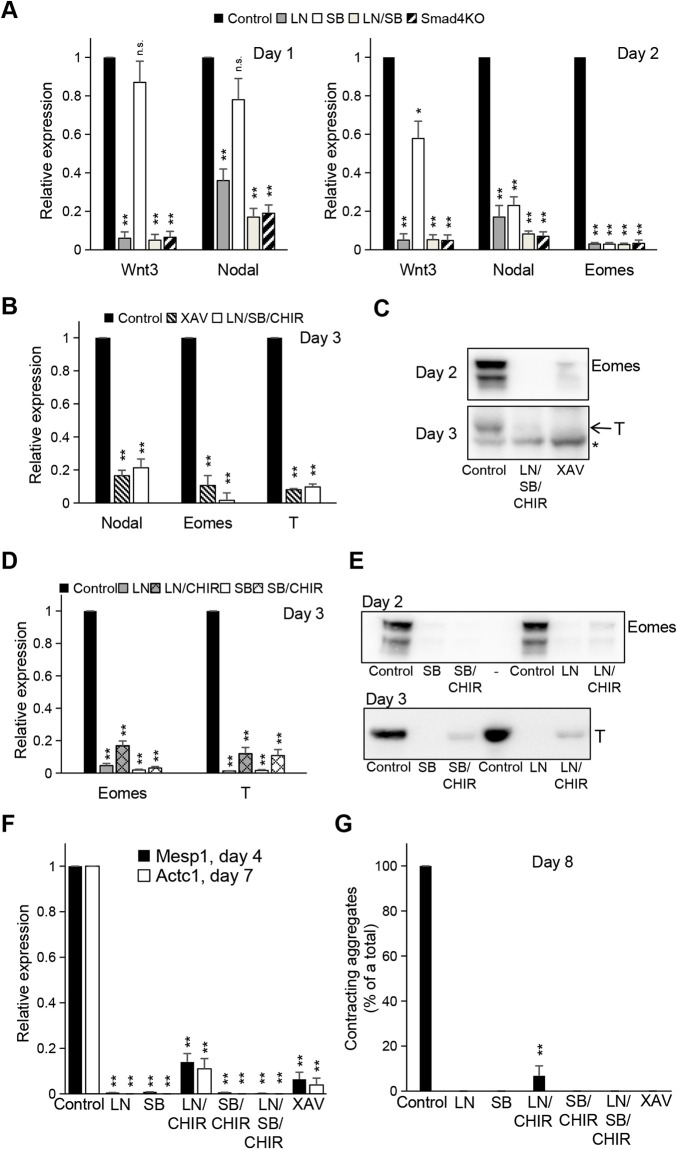
**Cardiac progenitor and cardiomyocyte formation require pSmad1/5, pSmad2/3 and WNT signaling.** (A) Barplots showing the expression of *Wnt3*, *Nodal* and *Eomes* in day 1 (left) and day 2 (right) ME WT cells (control) or in cells treated with 1 μM LDN193189 (LN), 10 μM SB431542 (SB), a combination of LN and SB, or in *Smad4* KO cells. For all qRT-PCR experiments total RNA was used, expression levels were calculated relative to the corresponding WT control (*n*=3). (B) Barplot displaying the expression of *Nodal*, *Eomes* or *T* in day 3 ME WT cells (control) or in cells treated with 10 μM XAV939 (XAV) or a combination of 1 μM LN, 10 μM SB and 5 μM CHIR99021 (LN/SB/CHIR) (*n*=3). (C) Western blot analysis of Eomes or T protein expressed in cells treated as in B. Asterisk indicates a background band. (D) Barplots of *Eomes* or *T* expression in day 3 ME WT cells (control) or in cells treated with 1 μM LN alone or with 5 μM CHIR (LN or LN/CHIR), with 10 μM SB alone or with 5 μM CHIR (SB or SB/CHIR). (E) Western blot analysis of Eomes or T protein expression in cells treated as in D. (F) Barplots showing the expression of *Mesp1* (day 4) or *Actc1* (day 7) in ME WT cells (control) or in cells treated with 1 μM LN alone or in combination with 5 μM CHIR (LN or LN/CHIR), with 10 μM SB alone or with 5 μM CHIR (SB or SB/CHIR), with a combination of 1 μM LN, 10 μM SB and 5 μM CHIR (LN/SB/CHIR), or with 10 μM XAV. (G) Barplot showing the fractions of contracting aggregates (% of total aggregates) at day 8 of differentiation. Cells were treated as in F and grown in 24-well plates. All aggregates in six wells were counted per treatment condition (*n*=6). See also Fig. S2. Data are mean+s.e.m. *P<5e-2, **P<1e-2 (paired two-tailed Student's *t*-test). n.s., not significant.

Next, we addressed the effects of BMP4 and Nodal signaling on *Wnt3* and *Nodal* induction by selectively inhibiting the Smad1/5 pathway with LDN193189 (LN), or the Smad2/3 pathway with SB431542 (SB) (Fig. S2A). *Wnt3* expression was abolished in LN-treated cells 1 day after BMP4 induction, whereas SB treatment reduced *Wnt3* levels only on day 2 (Fig. 2A). This result is corroborated by the observation that nuclear accumulation of Ctnnb1 on day 2 is reduced in LN- but not SB-treated cells (Fig. S2B). Our data is in agreement with the finding that recombinant Bmp4 is sufficient to induce *Wnt3* in isolated embryonic epiblast explants (Ben-Haim et al., 2006). We conclude that *Wnt3* is induced by Bmp4, possibly via the pSmad1/5 binding enhancer located in the second intron of the *Wnt3* gene (Fig. 1F).

Similarly, *Nodal* expression is reduced in LN-treated cells on day 1 and further on day 2. In contrast, in SB-treated cells, *Nodal* is strongly reduced only on day 2 (Fig. 2A). Wnt3 has been shown to enhance *Nodal* expression in the epiblast (Ben-Haim et al., 2006). Accordingly, inhibition of WNT with XAV939 (XAV) resulted in reduced *Nodal* levels on day 3 (Fig. 2B). However, activation of WNT with CHIR99021 (CHIR) in the absence of SMAD signaling (LN/SB/CHIR) was not sufficient to restore *Nodal* expression (Fig. 2B). These data suggest that *Nodal* upregulation requires both BMP and WNT activity.

Both LN and SB treatment alone abolished *Eomes* expression on day 2, just as the *Smad4* KO did (Fig. 2A). Different inhibitors of BMP or Nodal signaling confirmed the effect (Fig. S2C). Thus, pSmad1/5 is required for *Eomes* expression. SB treatment did not affect pSmad1/5 or WNT activity (Fig. S2A,B). Therefore, our data confirm the previous finding that pSmad2/3 activity is essential for *Eomes* expression (Senft et al., 2018). Similarly, inhibition of WNT resulted in strongly reduced *Eomes* levels, whereas WNT activation upon SMAD inhibition with LN/SB was not sufficient to rescue *Eomes* expression (Fig. 2B,C). Thus, WNT signaling is also required but not sufficient for *Eomes* induction. Furthermore, activation of WNT signaling by CHIR in the presence of LN only slightly restored *Eomes* expression (Fig. 2D,E; Fig. S2D). Together, these data show that WT levels of *Eomes* expression require BMP signaling via pSmad1/5 and WNT signaling, in addition to pSmad2/3 activity.

Next, we assayed the effects of pSmad1/5, pSmad2/3 and WNT on *T* expression (Fig. 2B-E; Fig. S2D). Inhibition of either pathway alone resulted in strong downregulation of T on day 3, and WNT induction by CHIR was not sufficient to rescue T expression to WT expression levels in either LN- or SB-treated cells. Thus, proper *T* expression also requires pSmad1/5, pSmad2/3 and WNT signaling.

In line with these data, expression of the Eomes target and cardiac progenitor gene *Mesp1* (Saga et al., 2000), as well as of the cardiomyocyte-specific marker *Actc1*, were strongly downregulated by LN or SB treatment (Fig. 2F). However, LN/CHIR- or XAV-treated differentiated cells showed low levels of *Mesp1* and *Actc1* expression. Of note, none of these conditions yielded contracting aggregates on differentiation day 8, except for the control and for LN/CHIR treatment, the latter in about 10% of the colonies (Fig. 2G). This indicates that WNT signaling can partially compensate for the loss of BMP signaling in producing cardiac mesoderm, albeit at low efficiency compared with control conditions.

Together, our data demonstrate that activities of pSmad1/5 and WNT signaling are required for proper cardiac mesoderm induction and efficient differentiation into contracting cardiomyocytes, in addition to pSmad2/3.

### Smad TFs activate mesodermal enhancers

**Fig. 3.**
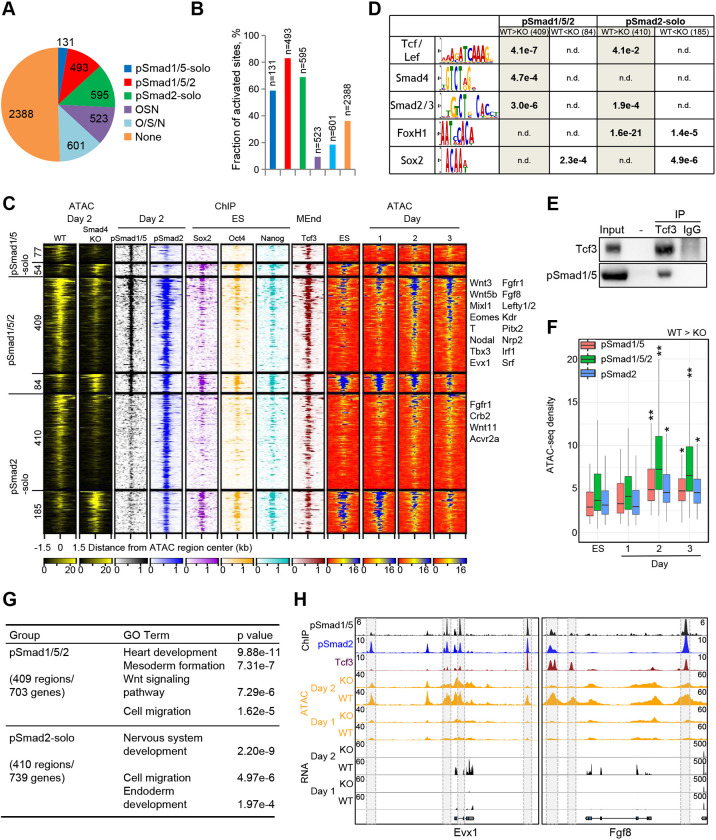
**Smad TFs activate mesodermal enhancers.** (A) Pie chart showing regions differentially accessible (DA) between WT and *Smad4* KO ME cells occupied by pSmad1/5 (Smad1/5-solo), pSmad1/5 and pSmad2 (pSmad1/5/2) or pSmad2 (pSmad2-solo) in day 2 ME cells, Oct4/Sox2 and Nanog (OSN), or any one or two of Oct4, Sox2 and/or Nanog (O/S/N) in ESCs, or none. (B) Barplot of the fractions of activated (WT>KO) DA regions in groups from A. (C) Clustered heatmaps of ATAC-seq reads in WT and *Smad4* KO day 2 ME cells, centered on DA regions, and corresponding heatmaps of ChIP-seq and ATAC-seq reads during differentiation. Clusters contain activated and repressed DA sites occupied by pSmad1/5-solo, pSmad2 or pSmad1/5/2. Selected genes bound by regions from corresponding clusters are listed on the right. (D) Motifs enriched in DA regions from the groups defined in C. n.d., not detected. (E) Western blot analysis of Tcf3 or pSmad1/5 in the input or eluates following immunoprecipitation with Tcf3 or IgG antibodies. (F) Boxplot of the quantification of normalized ATAC-seq density in groups containing activated DA sites defined in C during the differentiation course. *P<5e-6, **P<5e-10 (paired two-tailed Student's *t*-test). Boxplots show median values (middle bars) and first to third interquartile ranges (boxes); whiskers indicate 1.5× the interquartile ranges. (G) GO terms enriched among genes associated with activated pSmad1/5/2 or pSmad2-occupied DA regions. (H) Snapshots of pSmad1/5 or pSmad2 (day 2 ME), or Tcf3 (MEnd) ChIP-seq, ATAC-seq and RNA-seq tracks in WT or *Smad4* KO ME cells at selected genomic loci. Grey boxes highlight selected DA regions. See also Fig. S3 and Table S3.

To gain insight into the mechanism of SMAD-mediated transcriptional regulation, pSmad1/5 and pSmad2 binding sites were examined for the chromatin accessibility state in day 2 ME cells. ATAC-seq profiling revealed that sites bound by pSmad1/5 alone had higher accessibility than those bound by pSmad2 (Fig. S3A). However, the strongest correlation between pSmad binding and chromatin opening was observed at sites co-occupied by pSmad1/5/2 (Fig. S3A). To examine the effect of pSmad binding on global chromatin accessibility, we compared ATAC-seq profiles in WT and *Smad4* KO ME cells on day 2 and detected a total of 4731 differentially accessible (DA) regions (Fig. S3B). To quantify the occupancy of TFs at DA regions we defined six groups. We detected DA regions bound by pSmad1/5-solo, pSmad1/5/2 or pSmad2-solo. The remaining DA regions were grouped into those bound by Oct4, Sox2 and Nanog (OSN) in mESCs, by one or two of these factors (O/S/N), or by none of the factors (None) (Fig. 3A). The majority of pSmad1/5-solo, pSmad1/5/2 or pSmad2-solo bound regions, but only a small fraction of O/S/N or OSN bound regions, were activated by Smads (Fig. 3B). Notably, DA sites were mainly located at distal putative enhancer regions [more than 10 kb from the transcriptional start site (TSS)] (Fig. S3C). We then plotted the TF occupancy and ATAC-seq density at pSmad1/5-solo, pSmad2-solo and pSmad1/5/2 regions in WT and *Smad4* KO ME cells (Fig. 3C). Activated regions were strongly bound by the corresponding Smad TFs at day 2 of differentiation, repressed regions displayed an enrichment of pluripotency TF binding in mESCs, suggesting that Smads repress OSN-occupied sites upon differentiation (Fig. 3C; Fig. S3D).

To gain insight into the mechanism of SMAD-mediated opening of enhancers, we scanned the largest groups (Smad1/5/2 and Smad2-solo) for TF motifs and found that activated regions were enriched in SMAD and Tcf motifs, suggesting direct integration of TGFβ and WNT signaling (Fig. 3D). To substantiate this finding, we made use of Tcf3 ChIP-seq data derived from mesendodermal cells (Wang et al., 2017) and found that Tcf3 binding was strongly enriched at activated regions of the pSmad1/5/2 cluster (Fig. 3C; Fig. S3D). Notably, using immunoprecipitation we showed that pSmad1/5 and Tcf3 physically interact, suggesting that pSmad1/5 associates with Tcf3 at mesodermal and mesendodermal enhancers, and both cooperatively activate mesodermal gene expression (Fig. 3E).

We monitored the accessibility changes of SMAD-dependent DA regions by ATAC-seq on days 1 to 3 of the differentiation course (Fig. 3C). Activated loci exhibited low accessibility in ESCs and transiently opened during differentiation, reaching the peak on day 2, with the highest chromatin accessibility levels detected at pSmad1/5/2 sites (Fig. 3F). Compared with pSmad1/5/2 sites, the 410 pSmad2-solo sites displayed low levels of Tcf3 binding (Fig. S3D) and only a moderate increase in accessibility levels on day 2 (Fig. 3C,F). These sites were associated with 739 genes enriched in GO terms related to cell migration (49 genes), endoderm (9) and nervous system development (51) (Fig. 3G; Table S3). Most of the latter 51 genes were not expressed (Table S3), and the associated 52 sites displayed reduced accessibility compared with all other (358) pSmad2-solo regions (Fig. S3E). In contrast, genes associated with pSmad1/5/2 bound sites were enriched in mesoderm and heart development, and in WNT pathway GO terms (Fig. 3G). Genes coupled to activated Smad1/5/2/Tcf3 enhancers comprised nascent mesoderm control factors, such as *Eomes*, *T*, *Wnt3*, *Fgf8*, *Mixl1* and *Kdr*, strongly upregulated during early stages of differentiation (Fig. S1C) (Meilhac and Buckingham, 2018; Fig. 3C; Table S3). The comparison of active enhancer-close genes with pSmad1/5/2 direct targets revealed 63 SMAD-activated genes (Fig. S3F,G) related by GO analysis to mesoderm development, WNT signaling and heart morphogenesis terms, comprising many of the most strongly activated mesodermal targets of pSmad1/5/2, e.g. *Id1*, *Eomes*, *T*, *Pitx2*, *Mixl1*, *Fgf8*, *Evx1*, *Lefty1/2*, *Wnt3*, *Wnt5b*, *Tbx3*, *Axin2*, *Nodal*, *Irf1* and *Foxb1* (Fig. 3H; Fig. S3H,I; Table S3).

To summarize, we show that upon cardiac mesoderm induction with BMP4, pSmad1/5 forms a complex with Tcf3, which co-localizes with pSmad2 at mesodermal enhancers. These enhancers are associated with the strongest direct targets of pSmad1/5/2 and undergo a SMAD-dependent opening during the course of differentiation, correlated with activation of mesodermal gene expression.

### Cooperation of BMP, Nodal and WNT signaling is pivotal to opening of mesodermal enhancers

**Fig. 4.**
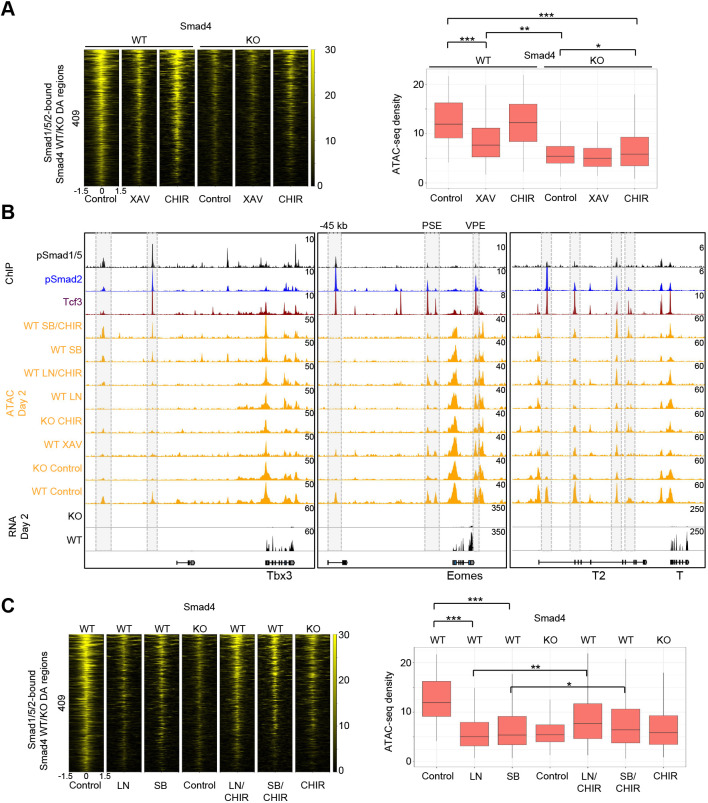
**Cooperation of BMP, Nodal and WNT signaling is pivotal to opening of mesodermal enhancers.** (A) Heatmap of ATAC-seq reads and boxplot showing quantification of normalized ATAC-seq density in WT and *Smad4* KO day 2 ME cells, centered on 409 differentially accessible (DA) regions occupied by pSmad1/5/2 as defined in Fig. 3C. Cells treated with 10 μM XAV (XAV), 5 μM CHIR (CHIR) and control cells are depicted. (B) Snapshots of pSmad1/5 and pSmad2 (day 2 ME) and Tcf3 (MEnd) ChIP-seq, ATAC-seq and RNA-seq tracks in WT or *Smad4* KO ME cells at selected genomic loci. Grey boxes highlight selected DA regions. (C) Heatmap of ATAC-seq reads and boxplot showing quantification of normalized ATAC-seq density in WT and *Smad4* KO day 2 ME cells, centered on the same regions as in A. Cells treated with 1 μM LDN193189 (LN) alone or in combination with 5 μM CHIR (LN or LN/CHIR), with 10 μM SB alone or together with 5 μM CHIR (SB or SB/CHIR), with 5 μM CHIR (CHIR) and with control cells are depicted. *P<5e-2, **P<5e-6, ***P<5e-10 (paired two-tailed Student's *t*-test). Boxplots show median values (middle bars) and first to third interquartile ranges (boxes); whiskers indicate 1.5× the interquartile ranges.

We then set out to determine the specific roles for BMP, Nodal and WNT signaling in the activation of mesodermal enhancers. We chose to monitor accessibility levels of 409 DA Smad4-dependent enhancers, which are co-occupied by pSmad1/5, pSmad2 and Tcf3, and display the strongest opening during mesoderm formation (Fig. 3C,F). To assess the effect of WNT signaling, we induced WT and *Smad4* KO cells with BMP4 followed by inhibition of the WNT pathway with XAV, 18 h post induction. Quantification of the ATAC-seq density revealed that WNT inhibition significantly reduced enhancer accessibility; however, not to the level of the *Smad4* KO (Fig. 4A,C). Activation of WNT with CHIR in *Smad4* KO cells increased enhancer accessibility, but was not sufficient to restore accessibility to WT levels. Thus, binding of WNT effectors to mesodermal enhancers contributes to their opening, but without SMAD signaling is not sufficient to establish average accessibility levels equal to WT. This result is in line with our data showing only partial rescue of cardiac gene expression and cardiomyocyte differentiation by WNT signaling in the absence of pSmad1/5 activity (Fig. 2B-G).

Furthermore, inhibition of either pSmad1/5 (LN) or pSmad2/3 (SB) resulted in closure of mesodermal enhancers, with average accessibility levels similar to *Smad4* KO (Fig. 4B,C). WNT activation in combination with individual pSmad1/5 (LN/CHIR) or pSmad2/3 (SB/CHIR) inhibition increased enhancer opening to some degree, but not sufficiently to achieve accessibility levels observed in control WT cells (Fig. 4B,C). Together, these results demonstrate that BMP and Nodal branches of SMAD signaling as well as WNT signaling are required for opening of mesodermal enhancers and subsequent induction of cardiac mesoderm.

### Upregulation of cardiac progenitor genes involves a positive feedback loop between Eomes and T

**Fig. 5.**
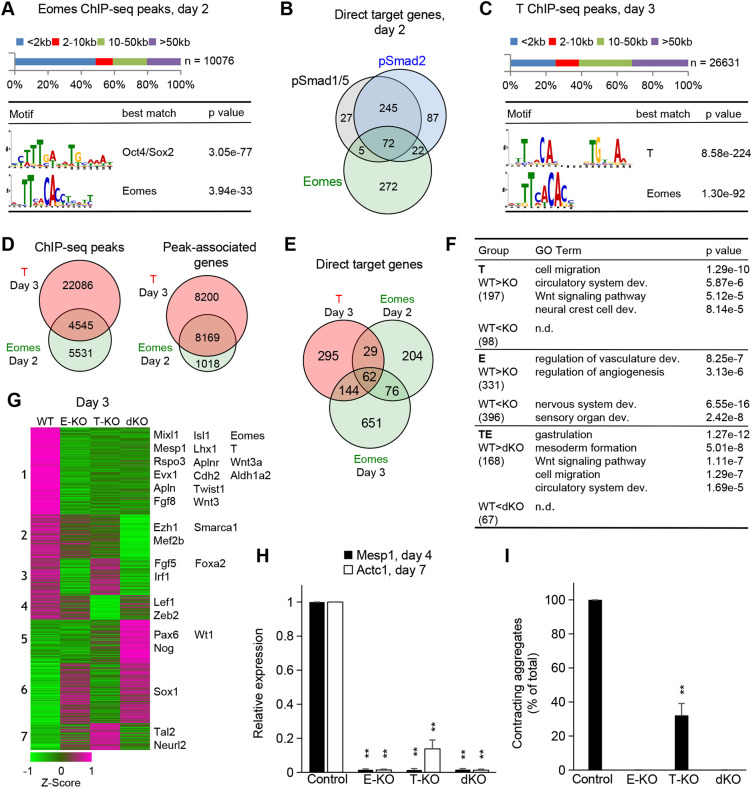
**Upregulation of cardiac progenitor genes involves a positive feedback loop between Eomes and T.** (A) Quantification of distances between Eomes binding sites in day 2 ME cells and nearest gene promoters (top). Top two *de novo* motifs discovered at Eomes binding sites (bottom). (B) Venn diagram displaying the overlap between the direct target genes of pSmad1/5, pSmad2 and Eomes at day 2. (C) Quantification of distances between T binding sites in day 3 ME cells and nearest gene promoters (top). Top two *de novo* motifs discovered at T binding sites (bottom). (D) Venn diagrams indicating the overlap between T and Eomes DNA binding sites or the overlap between genes bound by T and Eomes in ME cells. (E) Venn diagram showing overlap between T and Eomes direct target genes. (F) GO terms overrepresented in T, Eomes (E) and common (TE) day 3 direct target genes subdivided into activated (WT>KO) and repressed (WT<KO) groups. n.d., not detected. (G) K-means clustered heatmap representation of the expression of T and/or Eomes direct target genes in WT, *Eomes* KO (E-KO), *T* KO (T-KO) and double KO (dKO) day 3 ME cells. Selected genes from each cluster are listed on the right (RNA-seq, average of two replicates). (H) Barplot of the expression of *Mesp1* (day 4) and *Actc1* (day 7) in ME WT, E-KO, T-KO and dKO cells (*n*=3). (I) Barplot of the fractions (% of total) of contracting aggregates at day 8. Cells were treated as in H and grown in 24-well plates. All aggregates in six wells per treatment condition were counted. See also Fig. S4 and Table S3. Data are mean+s.e.m. **P<1e-2 (paired two-tailed Student's *t*-test).

Above we showed that WNT and Smads cooperatively induce *Eomes* and *T* expression. Similar to their expression in the posterior part of the PS, *Eomes* and *T* expression patterns overlap *in vitro*, with *Eomes* reaching its highest levels on day 2 and *T* on day 3 (Fig. S1H). To provide insight into Eomes function on day 2, we first examined its DNA binding pattern, using *Eomes* KO cells to detect and discard false-positive peaks (Table S3). Eomes binding sites were enriched in Oct4/Sox2 and Eomes (T-box) motifs, and approximately half the Eomes peaks (5168) were localized at promoter-far sites (Fig. 5A). In the posterior PS of the E7 embryo these sites are associated with H3K27Ac and H3K4me1 marks, suggesting that they represent active and/or poised enhancers (Yang et al., 2019; Fig. S4A). We identified 371 putative direct target genes of Eomes on day 2 (Fig. S4B). Among the activated targets were cardiac progenitor genes *Fgf8*, *Hand1*, *Nrcam*, *Mixl1*, *Apln* (Meilhac and Buckingham, 2018), and genes involved in cell migration and circulatory system development, whereas repressed targets included the neural regulators *Sox2*, *Pax6*, *Tal2* and *Sox1*, and were most significantly enriched in the GO term central nervous system development (Fig. S4C,D).

The overlap of activity levels, similarities in biological functions of direct target genes and enrichment of the Oct4/Sox2 motif in the binding sites suggested that in day 2 ME cells Smads and Eomes function cooperatively. In support, Eomes has been shown to physically interact with Smad1/5 and Smad2/3 in differentiated human (h) ESCs (Faial et al., 2015). Accordingly, Eomes peaks showed significant overlap with pSmad peaks, and the majority of pSmad1/5/2-bound genes also displayed Eomes binding (Fig. S4E,F). Yet, the putative direct targets of pSmad1/5, pSmad2 and Eomes showed only a moderate overlap on day 2 (Fig. 5B), whereas on day 3 most of the Smad1/5/2 direct targets were associated with Eomes binding and were downregulated in *Eomes* KO cells (Fig. S4G,H; Table S3). We suggest that this moderate overlap on day 2 might be due to compensation of the loss of Eomes by Smads on day 2, when SMAD activity is at its maximum.

Next, to gain insight into the role of T in early cardiac mesoderm formation, we examined its DNA binding pattern in day 3 ME, using *T* KO cells to detect and discard false-positive peaks (Table S3). We detected 26,631 binding sites, of which 19,869 (74%) are located outside the promoter at putative enhancers, as supported by the enrichment of active enhancer chromatin marks associated with a large fraction of sites occupied by T in the PS of E7.5 embryos (Yang et al., 2019; Fig. 5C; Fig. S4I). We detected 530 putative direct targets of T (Fig. S4J), most of which were positively regulated, suggesting that at day 3 of differentiation T mainly acts as an activator of transcription (Fig. S4K). Activated T targets are involved in mesoderm development, WNT signaling, cell migration and circulatory system development, and include cardiac progenitor genes, e.g. *Eomes*, *Fgf8*, *Mixl1*, *Rspo3*, *Mesp1*, *Apln*, *Evx1*, *Lhx1*, *Isl1* and *Aldh1a2* (Fig. S4K,L).

Apart from the palindromic T motif, T peaks were also strongly enriched in the Eomes motif (Fig. 5C). The comparison of their DNA occupancy revealed that 4545 (45%) of Eomes day 2 peaks were also occupied by T on day 3 (Fig. 5D). Moreover, 8169 (88%) of Eomes-bound genes were also bound by T. We found 91 direct targets that were common between Eomes on day 2 and T on day 3, and another 144 on day 3 (Fig. 5E). Unique T or Eomes targets were associated with distinct but related functions (Fig. 5F). Genes co-activated by T and Eomes play a role in gastrulation, mesoderm formation, cell migration, WNT signaling pathway and circulatory system development. These common direct targets comprise most of the cardiac mesoderm regulators, among them Mesp1, the expression of which depends on both Eomes and T (Fig. S4H). Our data indicate that in day 3 ME cells, Eomes and T function mostly cooperatively, but have additional separate roles.

To address the mechanism of cooperative target gene regulation, we identified DE genes between day 3 *Eomes*/*T* double KO (dKO) and WT cells. T- and Eomes-bound DE genes in dKO cells (1018 genes) were grouped in seven clusters based on their expression patterns in WT, single and double KO cells (Fig. 5G; Table S3). Genes in clusters 3 and 6 are regulated by Eomes alone. Clusters 2, 4, 5 and 7 contain genes that are similarly either activated or repressed by Eomes or T. Repressed genes in cluster 5 include the neural fate marker *Pax6* and the Bmp4 inhibitor *Nog*. Cluster 1 genes require the activity of both Eomes and T, as either KO alone causes downregulation relative to WT to a similar degree as observed in dKO. These include most of the crucial cardiac progenitor genes, such as *Mesp1*, *Eomes*, *T*, *Mixl1*, *Fgf8*, *Wnt3*, *Evx1*, *Isl1* and *Aldh1a2.* In contrast, *Foxa2*, expressed in a small subpopulation of early cardiovascular progenitors as well as other cell types, is regulated by Eomes alone, in line with roles for Eomes and Foxa2 in endodermal lineage specification (Burtscher and Lickert, 2009; Bardot et al., 2017; Fig. 5G). Of note, we show here that the cardiac lineage TF Mesp1 requires both Eomes and T for its full activation (Fig. 5G,H). Importantly, Eomes and T directly upregulate each other in day 2 and day 3 ME cells (Fig. 5G; Fig. S4M). Nevertheless, downregulation of Eomes in *T* KO cells is not complete (Fig. S4M), and differentiated *T* KO cells express *Actc1*, but at a low level, and only 30% of the colonies are contracting on day 8 of differentiation, suggesting that cardiac mesoderm formation is affected by loss of T (Fig. 5H,I).

In summary, we show that in Bmp4-induced cardiogenic mesoderm Eomes activates and forms a positive feedback loop with T. They control partially overlapping sets of target genes. Our data confirm the pivotal role of Eomes for proper cardiac mesoderm development, but in addition suggest an important novel role for T, as major cardiac progenitor genes including *Mesp1* require both TFs for WT expression levels, and the *T* KO impairs cardiomyocyte development *in vitro*.

### Eomes and T cooperatively activate mesoderm enhancers

**Fig. 6.**
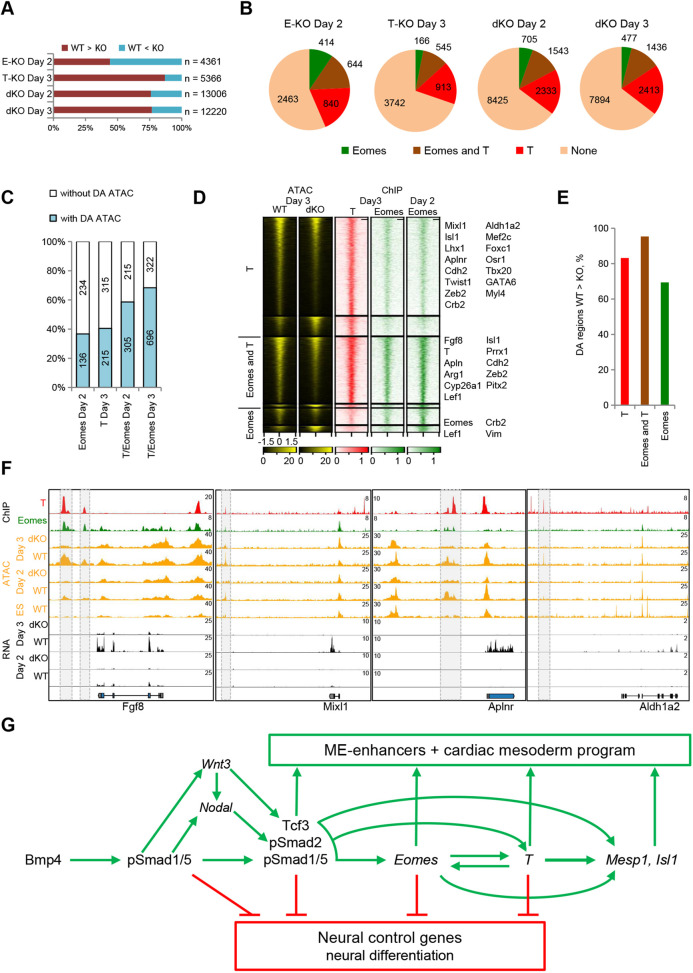
**Eomes and T cooperatively activate mesoderm enhancers.** (A) Quantification of the fractions of activated (WT>KO) or repressed (WT<KO) differentially accessible (DA) regions in *Eomes* KO (E-KO), *T* KO (T-KO) and double KO (dKO) ME cells. (B) Pie charts showing occupation of DA regions between WT and E-KO, T-KO or dKO ME cells by Eomes, T or both. (C) Barplot of the fractions and number of Eomes, T, or T/Eomes direct targets bound by regions DA between WT and E-KO, T-KO or dKO ME cells. (D) Clustered heatmaps of ATAC-seq reads in WT or dKO day 3 ME cells, centered on DA regions, and corresponding heatmaps of ChIP-seq reads. Clusters contain activated or repressed DA sites occupied by T, Eomes or both. Selected genes bound by regions from corresponding clusters are listed on the right. (E) Barplot showing fractions of activated (WT>KO) DA regions between WT and dKO day 3 ME cells occupied by T, Eomes or both. (F) Snapshots of ChIP-seq, ATAC-seq and RNA-seq tracks in WT or dKO ME cells at selected loci. Gray boxes highlight selected DA T- and/or Eomes-bound enhancers. (G) Schematic model of the regulatory network orchestrating cardiac mesoderm formation. See also Fig. S5 and Table S3.

Recent studies have shown that T is required for chromatin remodeling of neuro-mesodermal progenitors (NMPs), and that Eomes and T open chromatin in mesendodermal cells (Tosic et al., 2019; Koch et al., 2017). To examine whether Eomes and T remodel chromatin in early cardiac lineage cells, we performed ATAC-seq in *Eomes* KO, *T* KO and dKO cells. Individual ablation of *Eomes* or *T* resulted in 4361 or 5366 DA regions, respectively, whereas dKO changed the accessibility of more than 12,000 sites, emphasizing Eomes/T cooperation (Fig. 6A; Fig. S5A-D). A large fraction of the DA regions was bound by Eomes and/or T, suggesting that their accessibility is controlled by these TFs directly (Fig. 6B). Notably, the vast majority of activated DA sites were located outside of gene promoters at putative distal (>10 kb) enhancer regions (Fig. S5A-D). Thus, Eomes and T cooperatively regulate the accessibility of a set of putative enhancers, of which the majority become active.

To assess the functional relevance of Eomes/T-mediated enhancer accessibility regulation, we determined the fraction of direct target genes located next to DA sites. Although 37% (136 out of 370) of Eomes and 41% (215 out of 530) of individual direct T targets were located next to DA regions, for common targets there was an increase to 59% (dKO day 2; 305 out of 520) on day 2 and to 68% (dKO day 3; 696 out of 1018) on day 3, further supporting the finding of Eomes/T cooperation, in particular in day 3 ME cells (Fig. 6C). The majority of DA regions occupied by Eomes and/or T displayed positive regulation (Fig. 6D,E). Among the genes associated with the activated enhancer regions were cardiac progenitor (*T*, *Fgf8*, *Mixl1*, *Nanog*, *Lhx1*, *Lef1*, *Apln* and *Isl1*) and EMT (*Twist1*, *Prrx1*, *Crb2*, *Cdh2* and *Zeb2*) genes (Fig. 6D; Table S3). Of note, activated enhancers occupied by T were associated with markers of further differentiated cardiac mesoderm (*Gata6*, *Tbx20*, *Myl4*), suggesting that T might poise these enhancers for subsequent activation (Fig. 6D). While most of the T or Eomes activated regions are already open in ESCs, they undergo transient T and/or Eomes-dependent increase in accessibility during differentiation (Fig. 6F; Fig. S5E,F). Thus, in early cardiac progenitor cells, the majority of direct Eomes and T target genes, including cardiac progenitor genes, are regulated by individual or cooperative activation of enhancers by T and/or Eomes.

## DISCUSSION

Here, we present the regulatory network controlling the first stages of cardiac mesoderm formation induced by Bmp4 in murine ESCs (Fig. 6G). We show that the Bmp4 effectors Smad1/5 are the prime TFs initiating the process, comprising a double feedforward mechanism involving WNT and Nodal signaling followed by *Eomes* activation, and a positive feedback loop between Eomes and T, finally resulting in activation of *Mesp1* and the cardiac lineage program.

In the mouse embryo, cardiac mesoderm specification is initiated in epiblast cells located in the proximal PS, which are exposed to BMP4 signals secreted by the neighboring extra-embryonic ectoderm and express low Nodal activity. Bmp4 and Nodal activate Smad1/5 and Smad2/3, respectively, which form active TFs by hetero-trimerization with Smad4. Although Smad4 plays a central role in TGFβ signaling *in vitro*, its requirement for cardiac mesoderm formation in mouse embryos has been challenged (Chu et al., 2004; Sirard et al., 1998; Xu et al., 2019). Here, we show that cells lacking *Smad4*, irrespective of induction by Bmp4 or by ActA, fail to express mesodermal factors *in vitro* (Fig. 1E; Fig. S1L). Furthermore, *Smad4* ablation greatly reduced DNA binding of both pSmad1/5 and pSmad2 (Fig. S1I). These results underline the essential role of *Smad4* in BMP and Nodal signaling during cardiac mesoderm formation. In agreement with our results, *Smad4* has also been shown to be required for cardiac differentiation in hESCs (Xu et al., 2019). In sharp contrast, cardiac lineage specification and formation of a rudimentary heart structure was not prevented by conditional Sox2-driven depletion of *Smad4* in the mouse epiblast (Chu et al., 2004). However, Smad4 loss in mutant embryonic cardiac mesoderm has not been checked in this experiment, leaving the possibility that the conditional KO was not sufficiently effective. Alternatively, the discrepancy between our *in vitro* and the *in vivo* data might be explained by the fact that the PS of conditional *Smad4* KO embryos expresses *T*, indicating that WNT signaling is active in the mutant (Chu et al., 2004), in line with the expression of *T* in *Smad1/5* double KO cells upon induction of WNT signaling (Fernandes et al., 2016). *Eomes* expression has not been examined in this context either. But as we have shown here, Eomes and T control each other in early cardiac mesoderm *in vitro*. Thus, it is conceivable that in the conditional *Smad4* mutant embryo T might activate Eomes to sufficient levels allowing at least some cardiac mesoderm to form. Alternatively, T might be able to activate early cardiac mesoderm genes by itself and partially compensate for a possible lack of Eomes. The latter mechanism has been shown for ActA-induced mesendoderm (Tosic et al., 2019). In any case, the fact that *Smad4* KO embryos develop only a rudimentary heart indicates that loss of SMAD activity does have a dramatic effect on heart development *in vivo* (Chu et al., 2004).

The requirement of Nodal and Smad2/3 in cardiac mesoderm specification is well established, whereas the role of Bmp4-activated Smad1/5 has not been well understood, mainly due to the lack of double *Smad1/5* mutant embryos. Genetic studies have shown that Bmp4 activates WNT signaling in the PS *in vivo* (Ben-Haim et al., 2006). Similarly, in a previous study, Bmp4-treated ESCs formed posterior PS-like cells, accompanied by enhanced *Wnt3* and *Nodal* expression *in vitro* (Nostro et al., 2008). Using selective inhibitors for the two SMAD branches, we show here that it is pSmad1/5 that specifically activates expression of *Wnt3* and *Nodal* at the early stages of cardiac mesoderm specification (Figs 1F, 2A).

We observed that pSmad1/5 and pSmad2 co-localize with the WNT effector Tcf3 at enhancers of cardiac progenitor genes (Fig. 3C). It has been shown in hESCs that, upon PS induction, Smad2 interacts with Ctnnb1 (Funa et al., 2015), and that Smad2/3 and Tcf3 converge on regulatory regions of mesendodermal genes in mESCs (Wang et al., 2017). Moreover, Smad2/3-bound enhancer regions displayed opening during ESC differentiation (Senft et al., 2018; Xu et al., 2018). We report here that in Bmp4-induced mESCs pSmad1/5 physically interacts with Tcf3, and that the opening of mesodermal enhancers co-occupied by pSmad1/5/Tcf3 complexes coincides with pSmad1/5 and WNT activity (Figs 3E, 4A-C). We found that inhibition of BMP signaling by LN only led to partial opening of mesodermal enhancers, low level expression of *Eomes*, *T* and *Mesp1*, and very low efficiency of functional cardiomyocyte formation (Figs 2D-G, 4B,C). However, opening of mesodermal enhancers to WT levels, proper induction of the cardiac mesoderm fate and efficient formation of cardiomyocytes does not occur without concurrent pSmad1/5, pSmad2/3 and WNT activity (Figs 2D-G, 4A-C).

Previous approaches of *in vitro* differentiation of ESCs to cardiac mesoderm have employed ActA, Activin/BMP or WNT signaling (Tosic et al., 2019; Zhao et al., 2019; Kattman et al., 2011; Yoney et al., 2018; Kim et al., 2015). ActA has been shown to activate expression of *Wnt3* (Yoney et al., 2018), and induction of WNT signaling resulted in expression of ligands and receptors of both Nodal and BMP pathways (Kim et al., 2015). Thus, apparently all induction schemes involve all three pathways in one way or another.

Several enhancers controlling *Eomes*, in particular the proximal enhancers PSE and VPE, and an enhancer located 45 kb upstream of the *Eomes* promoter, have been identified in endoderm cells (Simon et al., 2017). PSE was dispensable, and VPE deletion resulted in markedly reduced *Eomes* expression, suggesting that optimal *Eomes* expression requires additional enhancers (Simon et al., 2017). In ME cells, we found that VPE was still accessible upon depletion of SMAD and/or WNT signaling (Fig. 4B). In contrast, the −45 kb enhancer, which displayed the strongest binding of pSmad1/5 and pSmad2 at the *Eomes* locus, was closed upon ablation of *Smad4* as well as by inhibition of pSmad1/5 or pSmad2/3 activity, and its accessibility was reduced upon inhibition of WNT signaling (Fig. 4B). Collectively, these findings suggest that in Bmp4-induced nascent mesoderm, pSmad1/5, pSmad2 and WNT signaling together activate *Eomes* transcription via the −45 kb enhancer.

*Eomes* plays essential roles in several embryonic and extra-embryonic tissues, and its specificity is conferred upon by context-dependent cooperation with other TFs (Russ et al., 2000; Arnold et al., 2008; Costello et al., 2011; Nowotschin et al., 2013; Alexanian et al., 2017). In the anterior PS, Eomes cooperates with Smad2/3 to promote mesendoderm and definitive endoderm formation (Costello et al., 2011; Simon et al., 2017; Chia et al., 2019). In hESC-derived mesoderm, Eomes physically interacts with Smad1/5 and Smad2/3 (Faial et al., 2015). Here, we show that in ME cells, pSmad1/5, pSmad2 and Eomes cooperate to promote the cardiac fate, and on day 2 SMAD activity is sufficient to compensate for the loss of *Eomes*, whereas on day 3 Eomes becomes essential (Fig. 5B; Fig. S4E-H). Therefore, pSmad1/5 and pSmad2/3, together with the effectors of WNT signaling, first activate *Eomes*, and then cooperate with Eomes to promote expression of mesoderm factors, such as *T*, *Fgf8*, *Evx1*, *Mixl1* and *Kdr*. This feedforward mechanism has also been referred to as a self-enabling mechanism of SMAD (Hill, 2016).

In the PS, *Eomes* expression precedes and partially overlaps with that of the mesoderm TF *T*. Opposite modes of Eomes/T interaction have been reported: in the posterior PS Eomes activates *T* (Simon et al., 2017; Pfeiffer et al., 2018), whereas in the anterior PS-derived endoderm Eomes represses *T* (Teo et al., 2011). A recent study demonstrated that in ActA-treated mESCs, Eomes induced the mesendodermal program, but upon *Eomes* ablation, *T* was upregulated and compensated for loss of *Eomes* (Tosic et al., 2019). In contrast, we show that in Bmp4-induced mesoderm cells, Eomes and T form a positive feedback loop (Fig. 5G; Fig. S4M). Further, we found that Eomes and T cooperate to open a large set of enhancers regulating cardiac progenitor genes (e.g. *Mesp1*, *Fgf8*, *Apln*, *Mixl1*, *Lhx1*, *Isl1*) and EMT genes (e.g. *Twist1*, *Zeb2*, *Cdh2*, *Crb2*) (Fig. 6A-F). Moreover, concurrent expression of *Eomes* and *T* is required to activate major cardiac mesodermal fate genes, including *Mesp1*, previously only shown to be regulated by either Eomes or T (Costello et al., 2011; Tosic et al., 2019; David et al., 2011; Fig. 5G,H). Conforming with the formation of abnormally looped heart structures observed in *T* mutant embryos (Concepcion and Papaioannou, 2014), we show that *T* KO cells expressed low levels of *Actc1*, and only 30% of the colonies contracted on differentiation day 8 (Fig. 5H,I). In agreement with this data, *T* ablation resulted in downregulation of *Eomes* expression, confirming the effect of T on *Eomes* expression (Fig. S4M). The combined data therefore support our conclusion that T and Eomes cooperate during cardiac mesoderm formation in a positive feedback loop. We propose that the latter is true also for early cardiac mesoderm formation in the embryo. Collectively, these findings provide a new facet of Eomes and T interaction in controlling differential mesodermal/endodermal lineage specification.

We show that in addition to promoting the cardiac fate in ME cells, SMAD signaling, Eomes and T also repressed neural fate genes, in particular *Sox1*, *Sox2*, *Otx2*, *Pax6* and *Tal2* (Figs 1E,F, 5F; Fig. S4D,L). It has been shown that during trunk development T promotes the mesodermal fate of NMP descendants and acts antagonistically to Sox2 driving the neural fate (Koch et al., 2017). In addition, neural fate suppression by Eomes and T has also been demonstrated in ActA-induced early ME differentiation of mESCs (Tosic et al., 2019). Therefore, Sox2 is antagonized by mesoderm-promoting TFs consecutively during development, starting with pSmad1/5/2 at the onset of gastrulation in the epiblast, followed by Eomes and T during head, trunk and tail development. Thus, activation of the mesodermal program and parallel suppression of the neural fate by mesoderm control genes take place throughout development.

Together, we show that Bmp4 induces cardiac mesoderm formation in murine ESCs, confirming a pivotal role for BMP in heart development in mouse. We present a model comprising a double feedforward mechanism involving Bmp4/Smad1/5, Wnt3/Tcf3 and Nodal/Smad2/3, which need to cooperate for opening mesoderm enhancers and effecting expression of mesodermal genes, including *Eomes* and *T*. The combined action of all three signaling pathways and their target mesoderm control factors Eomes and T is needed for cardiac lineage specification and proper cardiac mesoderm development. None of the factors can be omitted without affecting cardiac mesoderm formation *in vitro*, suggesting that heart development in the embryo employs the same mechanisms. In sum, our data provide a detailed mechanistic model of the regulatory network controlling cardiac mesoderm formation in mouse (Fig. 6G).

## MATERIALS AND METHODS

### Experimental model and subject details

Smad4 WT (conditional alleles) and knock-out mESCs were generated from mice kindly provided by Prof. Elizabeth Robertson (University of Oxford, UK). F1G4 mESCs were used to generate *Eomes*, *T* and *Eomes*/*T* dKO cell lines. All cell lines were regularly tested for mycoplasma contamination.

### Culture of mouse embryonic stem cells (mESCs)

All mESC lines were maintained on plates coated with 0.1% gelatin (Sigma-Aldrich; G1393) on a layer of mitotically inactive primary mouse embryo fibroblasts (feeder cells) in ESC growth medium: knockout Dulbecco's Modified Eagle's medium (DMEM) (Thermo Fisher Scientific; 10829018) supplemented with 2 mM L-glutamine (Lonza; BE17-605E), 15% (v/v) heat-inactivated fetal calf serum, 1% (v/v) non-essential amino acids (Thermo Fisher Scientific; 11140050), 0.1 mM β-mercaptoethanol (Sigma-Aldrich; M3148), 1% (v/v) nucleosides (Sigma-Aldrich; ES-008-D), 1% (v/v) penicillin and streptomycin (Lonza; DE17-603E) and 1000 U/ml LIF (Chemicon; ESG1107) at 37°C and 7.5% CO_2_.

### Generation of KO and reporter mESC lines

WT mESCs were from a F1G4 background (George et al., 2007). Conditional *Smad4* KO cells (Smad4CA) were provided by Prof. Elizabeth J. Robertson (Chu et al., 2004). To generate *Smad4* KO cells, the first exon of the *Smad4* gene flanked by loxP sites was excised by transient expression of Cre-recombinase in Smad4CA cells, followed by expanding single clones on 96-well plates and screening for homozygous clones by PCR using primers outside of the deleted region.

*Eomes* and *T* KO cell lines were generated using double-nicking CRISPR/Cas9 approach with Cas9(D10A) nickase mutant and two pairs of guide RNAs surrounding the promoter and the first exon of the corresponding gene (Ran et al., 2013). Single stranded oligonucleotides (Table S4) were annealed and cloned into the BbsI site of px335A_hCas9_D10A_G2P plasmid (gift from Dr Boris Greber, Max Planck Institute for Molecular Biomedicine, Muenster, Germany). WT F1G4 cells were transfected with a modified plasmid and transiently selected with 1 µg/ml puromycin (Gibco; 10130127) for 2 days. Single clones were picked 7-9 days after transfection, plated onto 96-well plates and screened for genomic DNA deletions by PCR using primers outside of the deleted region (Table S4). The dKO cell line was generated from *Eomes* KO cells using the same approach as for generation of the *T* KO cell line. The absence of Eomes and T at transcript and protein levels in the differentiated *Eomes* KO, *T* KO and dKO cells was confirmed using RT-PCR and western blotting, respectively.

A fluorescent reporter cell line was generated as previously described (Koch et al., 2017). In short, we used the mouse T BAC (RP24-530D23) modified by replacing the starting codon of the *T* gene by a reporter gene containing H2B-mCherry, followed by the rabbit β-globin polyadenylation signal (provided by Manuela Scholze-Wittler, Max Planck Institute for Molecular Genetics, Berlin, Germany). The modified T BAC was linearized, followed by random integration into F1G4 cells. Single clones with BAC integration were detected by PCR.

### *In vitro* differentiation

mESCs were deprived of feeder cells by dissociating in trypsin and passaging for four consecutive 25 min periods. After 24 h cells were dissociated, and 40,000 cell/ml cell suspension was plated onto tissue culture plates in 5 µl droplets using electronic multi-channel pipettes. Plates were then inverted and incubated for 24 h. Drops containing mESC aggregates [corresponding to differentiation day 0 or embryoid bodies stage (ES)] were then pooled, washed twice with DMEM (Thermo Fisher Scientific; 12491015) and resuspended in differentiation medium: 48% (v/v) DMEM/F12 (Thermo Fisher Scientific; 11320033), 48% (v/v) Neurobasal medium (Thermo Fisher Scientific; 21103049), 1% (v/v) 200 mM L-glutamine (Lonza; BE17-605E), 1% (v/v) penicillin and streptomycin, 1% (v/v) B27 (Gibco; 17504044), 0.5% (v/v) N2 (Gibco; 17502048), 0.5% (v/v) 7.5% bovine serum albumin (BSA) fraction V (Gibco; 15260037), 0.1 mM thioglycerol (Sigma-Aldrich; M1753) supplemented with 20 ng/ml mouse recombinant Bmp4 (R&D Systems; 5020-BP-010). To start differentiation, mESC aggregates resuspended in differentiation medium containing Bmp4 were immediately plated on fibronectin-coated plates (Calbiochem; 341631). Differentiation medium supplemented with Bmp4 was refreshed every 24 h.

Alternatively, for ActA treatment, drops containing mESC aggregates (corresponding to differentiation day 0 or ES) were pooled, washed twice with DMEM and resuspended in differentiation medium containing 20 ng/ml recombinant human/mouse/rat Activin A protein (R&D Systems; 338-AC-010).

For selective inhibition of Smad signaling pathways, the mESC aggregates were preincubated for 30 min with LN193189 (Sigma-Aldrich; SML0559), K0288 (Sigma-Aldrich; SML1307), SB431542 (Biogems; 3014193) or A-83-01 (Sigma-Aldrich; SML0788) at the indicated concentrations. Afterwards, the inhibitors we also added to the differentiation medium. For activation of inhibition of WNT signaling, CHIR99021 (TOCRIS; 4423) or XAV939 (Sigma-Aldrich; X3004) were added to the medium at 18 h of differentiation at the indicated concentrations.

### Antibodies

The antibodies used for western blotting were: Phospho-Smad1/5 [Cell Signaling Technology (CST), 9516; RRID: AB_491015], Phospho-Smad2/3 (CST, 3108; RRID: AB_490941), Smad1 (CST, 9743; RRID: AB_2107780), Smad2/3 (CST, 5339; RRID: AB_10626777), Eomes (Abcam, ab23345; RRID:AB_778267), T (rabbit polyclonal anti-Brachyury, custom), Tcf3 (Santa Cruz Biotechnology, sc-166411), GAPDH (CST, 5174), H3 (Abcam, ab1791; RRID:AB_302613), active Ctnnb1 (CST, 8814; RRID: AB_11127203). The antibodies used for immunoprecipitation were: Tcf3 (Santa Cruz Biotechnology, sc-166411; RRID: AB_2302942), normal mouse IgG (Santa Cruz Biotechnology, sc-2025; RRID: AB_737182). The antibodies used for ChIP were: Phospho-Smad1/5/9 (CST, 13820; RRID: AB_2493181), Phospho-Smad2 (CST, 18338; RRID: AB_2798798), Eomes (Abcam, ab23345), T (Santa Cruz Biotechnology, sc-17743; RRID:AB_634980). The antibodies used for immunofluorescence and flow cytometry were: Myl7 (Proteintech, 17283-1-AP; RRID: AB_2250998; 1:250 dilution), Actc1 (Proteintech, 66125-1-Ig; RRID: AB_2881524; 1:200), Tnnt2 (Abcam, ab209813; 1:200), donkey anti-mouse IgG, Alexa Fluor 488 (Thermo Fisher Scientific, A-21202; RRID: AB_141607; 1:500), donkey anti-rabbit IgG, Alexa Fluor 488 (Thermo Fisher Scientific, A-21206; RRID: AB_2535792; 1:500).

### Analysis of protein expression and phosphorylation levels by western blotting

Whole-cell extracts were prepared from mESC or differentiating cells using 1× Novex NuPAGE LDS sample buffer (Thermo Fisher Scientific; NP0007) supplemented with 100 mM DTT (Sigma-Aldrich; 43815). DNA was digested using Benzonase Nuclease (Millipore; E1014). Samples were heated at 95°C for 5 min, run on 4-12% NuPAGE Bis-Tris Protein Gel, transferred to nitrocellulose membrane, and subjected to western blotting using primary antibodies at 1:1000 dilution and the appropriate HRP-conjugated secondary antibodies at 1:5000 dilution. Detection was performed using Amersham ESL reagents (GE Healthcare) and scanned using a Fusion SL chemiluminescent detection system (Vilber). At least two biological replicates were performed for each experiment. For nuclear extraction, Nuclear Complex Co-IP Kit (Active Motif; 54001) was used following the manufacturer's instructions.

### Immunoprecipitation

Differentiated cells were collected and lyzed using Nuclear Complex Co-IP Kit. We added 2.5 μl primary Tcf3 antibody or mouse IgG antibody to the 300 μl of the nuclear fraction, followed by rotation overnight at 4°C. Then 50 μl protein G-coated Dynal beads were washed three times with PBS, resuspended in the nuclear fractions containing primary antibodies and rotated for 1 h at 4°C. The beads were then washed five times with 1 ml of the ice-cold IP washing buffer [50 mM Tris (pH 7.5), 150 mM NaCl, 1 mM EDTA, 1% Triton X-100], bound proteins were eluted using 100 μl 1× Novex NuPAGE LDS sample buffer and analyzed using western blotting.

### Fluorescence-activated cell sorting

To detect the percentage of cells expressing *T* during differentiation, we subjected F1G4 cells containing H2B-mCherry reporter to FACS analysis. Cells at various differentiation stages (ES/day 0, day 1, day 2, day 3, day 4 and day 5) were trypsinized, resuspended in 3% BSA in PBS and analyzed on a FACS Aria II (Becton Dickinson). For detection of cardiomyocytes expressing *Tnnt2*, day 6 differentiating cells were trypsinized and counted. Aliquots containing 2e6 cells were washed with 1 ml PBS, incubated with 2% formaldehyde in PBS for 10 min at room temperature and washed twice with 1 ml PBS. The aliquots were then incubated in 0.5 ml PBS containing 0.2% Triton X-100, followed by incubation with the primary Tnnt2 antibody for 60 min at room temperature. After three washing steps with 1 ml PBS/0.1% Triton X-100, cells were incubated with the fluorescently labeled secondary antibody for 30 min at room temperature. For the control experiment, primary Tnnt2 antibody was omitted. After three washing steps with 1 ml PBS/0.1% Triton X-100, cells were analyzed on a FACS Aria II.

### Immunofluorescence

For immunofluorescence, cells were washed twice with PBS and fixed in 4% paraformaldehyde in PBS for 10 min at room temperature while shaking, followed by two washes with PBS for 5 min and one wash with 0.1% Triton X-100 in PBS. Cells were then incubated for 1 h with 2% BSA in PBST (0.05% Tween 20 in PBS), rinsed with PBS, and incubated overnight with the primary antibodies diluted at 1:100 in PBST containing 0.2% BSA. After five washes with PBST, cells were incubated with the corresponding secondary antibodies (1:1000 dilution) and washed five times with PBS. The images were collected with ZEISS Axio Observer Z1 microscope.

### qRT-PCR analysis

For RNA extraction, cells were collected at the indicated times and processed with the RNeasy Micro kit (Qiagen). For each sample, 500 ng of total RNA was used for cDNA synthesis with QuantiTect Reverse Transcription Kit (Qiagen). Quantitative PCR was performed on a StepOnePlus Real-Time PCR System (Applied Biosystems) using *Pmm2* as the internal control gene for calculating relative expressions. Sequences of primers used for qRT-PCR are listed in Table S4. At least three biological replicates were performed for each experiment.

### RNA extraction and RNA-seq library preparation

RNA extraction and library preparation was performed as previously described (Koch et al., 2017). Briefly, total RNA was extracted from 40,000 mESCs or differentiated cells using Trizol reagent (Thermo Fisher Scientific; 15596026) following the manufacturer's instructions and purified using the RNeasy Micro kit (Qiagen). Residual genomic DNA was digested on a column. The RNA was quantified using Qubit RNA HS assay (Life Technologies), and the integrity of the RNA was assessed using Bioanalyzer RNA pico chips (Agilent).

Strand-specific RNA-seq libraries were generated from 100 ng of total RNA using the ScriptSeq v2 (Epicentre) low input library preparation kit according to the manufacturer's instructions. The library was amplified using 15 PCR cycles. RNA-seq libraries were quantified using the Qubit HS DNA assay (Life Technologies) and the size distribution was assessed using the DNA HS Bioanalyzer chips (Agilent). Libraries were pooled and paired-end sequenced on a HiSeq 2500 with 50 bp read lengths.

### Chromatin immunoprecipitation

ChIP experiments were carried out as previously described (Koch et al., 2017). Briefly, crosslinking was performed directly on differentiating cells in differentiation medium with the addition of 1/10 volume of crosslinking solution [11% formaldehyde, 50 mM Hepes (pH 7.8), 100 mM NaCl, 1 mM EDTA, 0.5 mM EGTA] for 10 min at room temperature, while shaking. The crosslinking reaction was quenched with the addition of 1/10 volume of 2.5 M glycine and 5 min incubation. Cells were washed twice with cold PBS, scraped in cold PBS containing 0.05% Triton X-100 and pelleted in aliquots of 5×10^7^. For sonication, complete protease inhibitors without EDTA (Roche) at 1× final concentration was added to all lysis buffers (LB) before use. Each pellet was resuspended in 2.5 ml LB1 (50 mM Hepes pH 7.5, 140 mM NaCl, 1 mM EDTA, 10% glycerol, 0.75% NP-40, 0.25% Triton X-100) and rotated at 4°C for 20 min. The cell suspension was homogenized using a douncer. The chromatin was pelleted by centrifugation at 1400 ***g*** and 4°C for 5 min and resuspended in 2.5 ml LB2 [10 mM Tris (pH 8), 200 mM NaCl, 1 mM EDTA, 0.5 mM EGTA]. After 10 min rotation at 4°C, the centrifugation step was repeated and each pellet was resuspended in 1.5 ml LB3 [10 mM Tris (pH 8), 1 mM EDTA, 0.5 mM EGTA, 100 mM NaCl, 0.1% Na-deoxycholate, 0.5% N-lauroylsarcosine], transferred to 15 ml Falcon tubes and sonicated using a W-450D Digital Sonifier (Branson) for 14 cycles of 10 s on/50 s off in a 4°C cold room with tubes chilled in ice water. After sonication, 150 μl of Triton X-100 was added per tube, transferred to two 1.5 ml Eppendorf tubes and debris was pelleted by centrifugation at 20,000 ***g*** at 4°C for 10 min. The solubilized chromatin was then pooled and mixed thoroughly. After taking 50 μl as an input control, the chromatin was distributed into 1.5 ml aliquots, snap frozen and stored at −80°C until use.

The input was reverse-crosslinked with the addition of 50 μl 2× elution buffer [100 mM Tris (pH 8), 20 mM EDTA (pH 8), 2% SDS] and 13-15 h incubation at 65°C. Then 100 μl of TE buffer were added, and RNA was digested using 0.2 μg/ml RNase A at 37°C for 2 h. Proteins were digested with the addition of 0.2 μg/ml proteinase K and incubation at 55°C for 2 h. The DNA was purified with two subsequent phenol:chloroform:isoamyl alcohol (25:24:1, pH 8) extractions and a subsequent MinElute (Qiagen) purification. The input DNA was quantified using a NanoPhotometer (Implen).

For ChIP, 100 μl protein G-coated Dynal beads (Life Technologies) were washed three times with 1 ml of blocking buffer (PBS, 0.5% BSA), resuspended in 500 μl of blocking solution containing 5 μg ChIP antibody and rotated overnight at 4°C. Beads were then washed three times with 1 ml of blocking buffer and resuspended in 100 μl blocking buffer. Chromatin equivalent to 5×10^7^ cells was added and rotated overnight at 4°C. The following day, six washing steps (nine for T) with 1 ml RIPA buffer [50 mM Hepes (pH 7.6), 500 mM LiCl, 1 mM EDTA, 1% NP-40, 0.7% Na-Deoxycholate] and one washing step with 1 ml of TEN [10 mM Tris (pH 8), 1 mM EDTA, 50 mM NaCl] were performed. The elution was performed in two subsequent steps using 100 μl of 1× elution buffer [50 mM Tris (pH 8), 10 mM EDTA, 1% SDS] and incubation at 65°C while shaking for 10 min each. The eluates were combined and incubated for 13-15 h at 65°C. Finally, 200 μl of TE buffer were added and the ChIP DNA was purified as described for the input above. ChIP DNA was quantified using the Qubit (Life Technologies) DNA HS assay.

### ChIP-seq library preparation

The ChIP-seq libraries were generated using the TruSeq ChIP library kit (Illumina) with the previously described modifications (Koch et al., 2017). After adapter ligation, 0.95× of AMPure XP beads (Beckman Coulter) were used for a single purification and the DNA was eluted using 14 μl of resuspension buffer (RSB, Illumina). After the addition of 1 μl primer mix (25 mM each, Primer 1: 5′-AATGATACGGCGACCACCGAG-3′; Primer 2: 5′-CAAGCAGAAGACGGCATACGAG-3′) and 15 μl 2× Kapa HiFi HotStart Ready Mix (Kapa Biosystems), amplification was performed for 45 s at 98°C, five cycles of 15 s at 98°C, 30 s at 63°C and 30 s at 72°C, and a final 1 min incubation at 72°C. The PCR products were purified using 0.95× of beads and eluted using 21 μl of RSB. Ligation products were then separated using a 1.5% agarose gel. Post-run staining was performed using SYBR Gold (Life Technologies) under agitation for 30 min. Gel slices corresponding to ∼250-400 bp fragment size were cut out using a Dark Reader (Clare Chemical Research) transilluminator. The gel extraction was performed using five gel volumes of QG buffer (Qiagen) with the addition of one gel volume of isopropanol. The MinElute (Qiagen) columns were washed once with QG buffer and twice with PE buffer, air-dried for at least 10 min and eluted using 21 μl of EB buffer. We used 19 μl of the eluate in the final amplification, with the addition of 1 μl primer mix and 20 μl 2× Kapa HiFi HotStart premix. The same protocol as for the pre-amplification was used, with the exception of using 13 amplification cycles. The libraries were quantified using the Qubit DNA HS assay and the library size was validated using DNA HS Bioanalyzer chips. Sequencing was performed on either the HiSeq 2500 or NextSeq 500 (Illumina) using 2×50 bp or 2×75 bp read lengths, respectively.

### ATAC-seq

ATAC-seq was performed as previously described (Buenrostro et al., 2013). The cells were trypsinized, and the trypsin was inactivated by washing with cold PBS containing 2% BSA. For each sample, 50,000 cells were collected, washed with 1 ml cold PBS and pelleted by centrifugation (300 ***g***). The cells were lysed in 50 μl of cold lysis buffer [10 mM Tris (pH 7.4), 10 mM NaCl, 3 mM MgCl_2_, 0.1% IGEPAL CA-630] and immediately centrifuged at 500 ***g*** at 4°C for 10 min. The pellet was resuspended in the transposition reaction mix (25 μl 2× TD buffer, 2.5 μl Tn5 transposase, 22.5 μl H_2_O) and incubated at 37°C for 30 min. After incubation, the reaction was stopped with the addition of PB buffer (Qiagen) and the tagmented DNA was purified using the MinElute kit (Qiagen). The DNA was combined with the ATAC index PCR primers and 2× Kapa HiFi HotStart Ready Mix and pre-amplified [98°C 30 s, 5× (98°C 10 s, 63°C 30 s, 72°C 1 min)] in a 50 μl volume. To determine the remaining cycles to avoid potential over-amplification, 5 μl of the pre-amplification mix was combined with the primers, 1× Evagreen SYBR green (Jena Biosciences) and 2× Kapa HiFi HotStart Ready Mix in a 15 μl total volume and run for 30 cycles on a StepOne Plus. The remaining 45 μl of pre-amplified samples were amplified for a further 6-7 cycles and the libraries were purified using a MinElute column (Qiagen). The libraries were quantified using the Qubit DNA HS assay and the library sizes were validated using DNA HS Bioanalyzer chips. Samples were pooled and paired-end sequencing was performed on either the HiSeq 2500 or HiSeq 4000 (Illumina) using 2x 50 bp or 2×75 bp read lengths, respectively.

### Genome assembly

All datasets were mapped to the mouse mm10 reference genome containing chromosomes 1-19, X, Y, and M.

### Analysis of RNA-seq data

RNA-seq reads were subjected to quality control using FastQC (https://www.bioinformatics.babraham.ac.uk/projects/fastqc/) and trimmed using cutadapt (version 2.9) (https://doi.org/10.14806/ej.17.1.200) to remove adapter sequences. Alignment was performed using Hisat2 (Kim et al., 2019) (version 2.1.0) with the following arguments: ‘--no-mixed --no-discordant’. The index files for Hisat2 were built using mm10 reference genome containing chromosomes 1-19, X, Y and M, and splice sites and exon data from refSeq annotations. The resulting sam format files were then converted to bam format, sorted and indexed using samtools (Li et al., 2009) (version 1.9). FPKM values were calculated using Cuffdiff (Trapnell et al., 2010) (version 2.2.1) with the following arguments: ‘-u -no-effective-length-correction -b’. To identify genes that showed differential expression upon depletion of Smad4, Eomes or T, normalized FPKM values were filtered, and only genes with FPKM >1 in at least one of the samples were examined. Log_2_ fold change values were calculated using normalized FPKM comparing WT with KO, and genes with log_2_ fold change <1 or <−1 were selected.

For visualization, the reads from biological replicates were combined, and the data were normalized and converted to bigWig (bw) format using bamCoverage tool from deepTools (Ramirez et al., 2016) (version 3.1.3) with the following arguments: ‘--normalizeUsing BPM -bs 5 –ignoreDuplicates’. Genome browser snapshots containing RNA-seq data were generated using pyGenomeTracks (Ramirez et al., 2018) (version 3.1). The expression data were visualized in heatmaps using heatmap.2 function of gplots package (version 3.0.1) (https://CRAN.R-project.org/package=gplots) in R.

For clustering of genes regulated by T and/or Eomes, genes bound by T and DE in day 3 *T* KO compared with day 3 WT cells were combined with genes bound by T and DE in *Eomes*/*T* dKO cells compared with day 3 WT cells, resulting in a gene list of 1018 genes. The k-means clustering into seven groups was performed using Cluster3.0 (De Hoon et al., 2004), and visualized using Java TreeView (Saldanha, 2004).

### Analysis of ChIP-seq data

For 75 bp read length ChIP-seq, the reads were first trimmed to 50 bp length. The reads were then aligned to the reference genome using bowtie2 (Langmead and Salzberg, 2012) (version 2.2.4) with the following arguments: ‘-−3 5 -I 100 -X 500 --no-discordant --no-mixed’. Sam files containing mapped paired-end reads were converted to bam files, sorted and indexed using samtools. For visualization, the data were normalized and converted to bw format using bamCoverage tool from deepTools with the following arguments: ‘--normalizeUsing CPM --extendReads 200 --smoothLength 9 -bs 3 -ignore chrM’. Peak detection was performed using MACS2 (Zhang et al., 2008) (version 2.1.1.20160309) with q-value cutoffs of 1e-4 (for T), 5e-4 (pSmad1/5/9), 1e-7 (pSmad2), 5e-4 (Eomes day 2 and day 3) and 1e-2 for all TFs in the corresponding KO cells. MACS2-generated q-values were used to assess the peak-calling confidence. Genome browser snapshots containing ChIP-seq data were generated using pyGenomeTracks. ChIP-seq data from public repositories were aligned, and bw files were generated as described above. For *Sox2*, *Oct4* and *Nanog* ChIP-seq data, peaks were called using MACS2 with q value cutoff of 1e-4.

To identify genes potentially regulated by TF binding sites, we first overlapped the ChIP-seq peak summits of every TF with promoters (transcription start sites +/−2 kb) of refSeq annotated genes using bedtools (Quinlan and Hall, 2010) (version 2.27.1). This identified genes containing promoter-associated peaks. The remaining peak summits were associated to the genes with the closest up- and downstream promoters. These genes were combined with the genes containing promoter-associated peaks to form a total list of genes bound by a TF. A subset of TF-bound genes that displayed differential expression between WT and the corresponding TF KO cells were called putative direct target genes of the corresponding TF.

For comparison of binding sites of various TFs, ChIP-seq peaks were called overlapped if peak summit distances were less than 300 bp. Distribution of peaks based on distances from the nearest promoters based on peak summit and TSS coordinates was calculated using bedtools.

For *de novo* motif discovery within ChIP-seq peaks, we extracted genomic sequences in 100 bp (+/−50 bp) regions around peak summits using bedtools and used the resulting fasta files as input for Meme (version 5.1.1) in the MEME suite (Bailey et al., 2009). To find known motifs enriched within defined subsets of ChIP-seq peaks, such as peaks overlapping with the differentially accessible ATAC-seq regions, genomic sequences in 120 bp (+/− 60 bp) regions around corresponding peak summits were used as input for MEME-chip in the MEME suite.

Clustered ChIP-seq heatmaps were generated using computeMatrix and plotHeatmap tools from deepTools. Grouping into clusters was performed based either on peak overlaps of various TFs or on appropriate clusters of ATAC-seq regions. Coordinates of peak summits within each cluster and normalized bw ChIP-seq files were used as input. Boxplots indicating distributions of ChIP-seq densities within heatmap clusters were generated using ggplot2 (version 3.1.1, https://ggplot2.tidyverse.org/) package in R. The values were obtained from the output file of ‘—outFileNameMatrix’ argument of the computeMatrix tool run with additional argument ‘-bs 1500’*.*

### Analysis of ATAC-seq data

All ATAC-seq experiments were performed with two biological replicates. ATAC-seq reads were trimmed to 40 bp length and adapter sequences were removed using cutadapt. The trimmed reads were aligned to the reference genome using bowtie (Langmead et al., 2009) (version 1.1.2) with the following arguments: ‘-y -v 2 --best --strata -m 3 -k 1 -S -X 2000 --allow-contain’. Sam files containing mapped paired-end reads were converted to bam files, sorted and indexed using samtools. Reads mapped to chromosomes M and Y or known ATAC artifact regions (ENCODE) were removed using samtools. Possible PCR duplicates were removed using Picard (version 1.103, https://broadinstitute.github.io/picard).

For visualization, the reads from biological replicates were combined, and the data were normalized and converted to bw format using bamCoverage tool from deepTools with the following arguments: ‘--normalizeUsing RPCG --effectiveGenomeSize 2652783500 --extendReads 200 --smoothLength 9 -bs 3 -ignore chrY chrM’. All accessible regions were detected as narrowPeaks using MACS2 with q value cutoffs of 5e-2.

To identify regions that are differentially accessible between WT and KO cells, we used DiffBind package (version 2.12.0, https://bioconductor.org/packages/release/bioc/html/DiffBind.html) in R. As input, for each replicate of WT and KO ATAC-seq data, bam files containing filtered mapped reads and a list of genomic coordinates of accessible regions were used. DiffBind was run with default parameters, except the consensus peak list was set to include peaks detected in at least two of the four samples.

Detected ATAC-seq regions were considered to overlap with ChIP-seq peaks if the summit of a ChIP-seq peak was located within an ATAC-seq region. Clustered ATAC-seq heatmaps, with clustering based on the differential accessibility and/or overlap with TFs, were generated using computeMatrix and plotHeatmap tools from deepTools. Normalized ATAC-seq bw files and coordinates of ATAC-seq region centers were used as input. Boxplots indicating distributions of ATAC-seq densities within heatmap clusters were generated using ggplot2. The values were obtained from the output file of ‘—outFileNameMatrix’ argument of the computeMatrix tool run with additional argument ‘-bs 1500’.

### GO term analysis

GO term biological process enrichment analyses were performed using the PANTHER classification system (Mi et al., 2019) (version 14.0).

### Statistical analysis

Statistical significance of qRT-PCR as well as ATAC-seq and ChIP-seq density differences was assessed using a paired two-tailed Student's *t*-test as indicated in figure legends.

## Supplementary Material

10.1242/develop.201450_sup1 Supplementary information
