# Supplementary material for: BMP4 triggers regulatory circuits specifying the cardiac mesoderm lineage
Source: Development. 2023 May 22;150(10):dev201450. doi: 10.1242/dev.201450 (PMC10233716; doi:10.1242/dev.201450)
Supplement: Supplementary information [file develop-150-201450-s1.pdf]

Figure S1

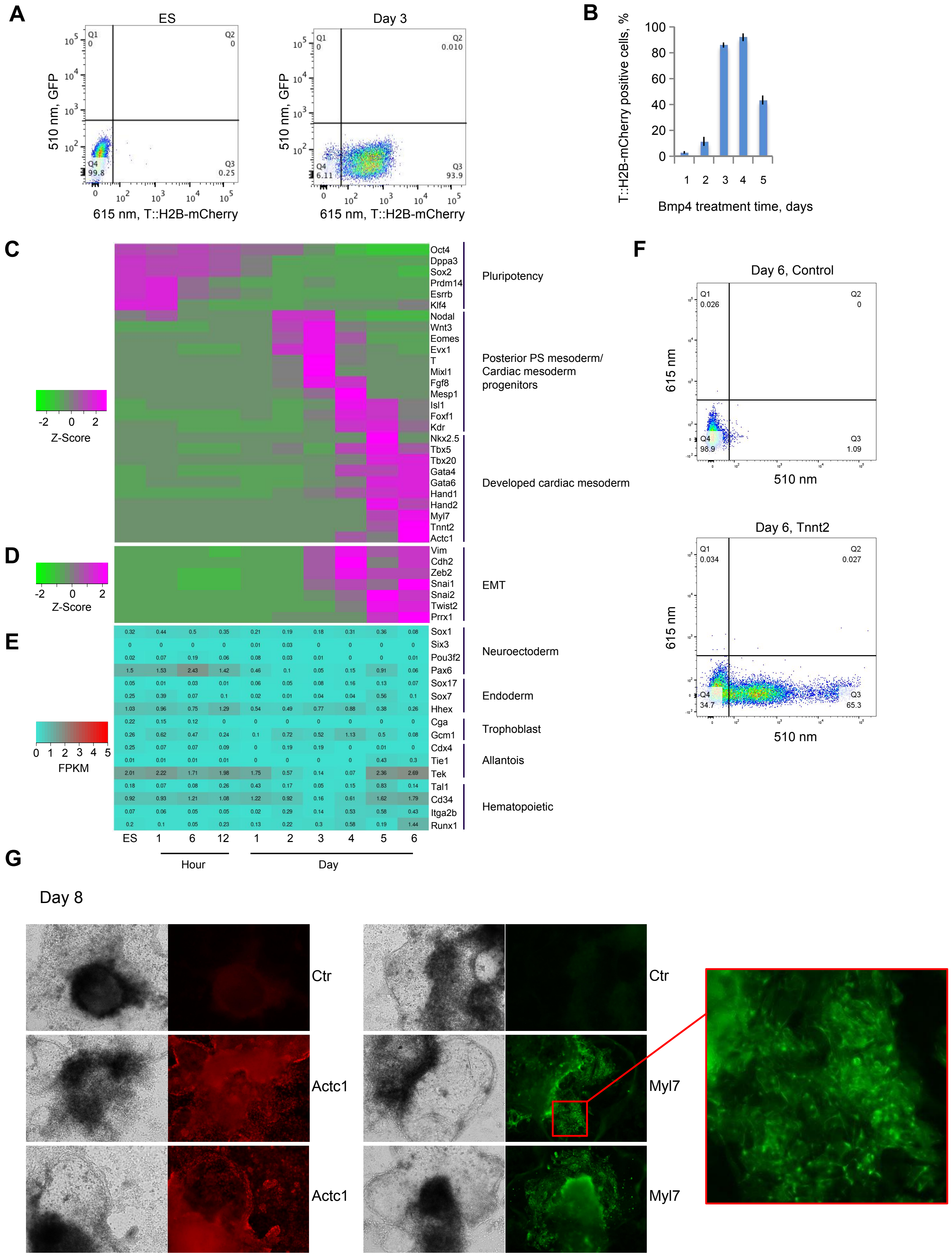

Development • Supplementary information

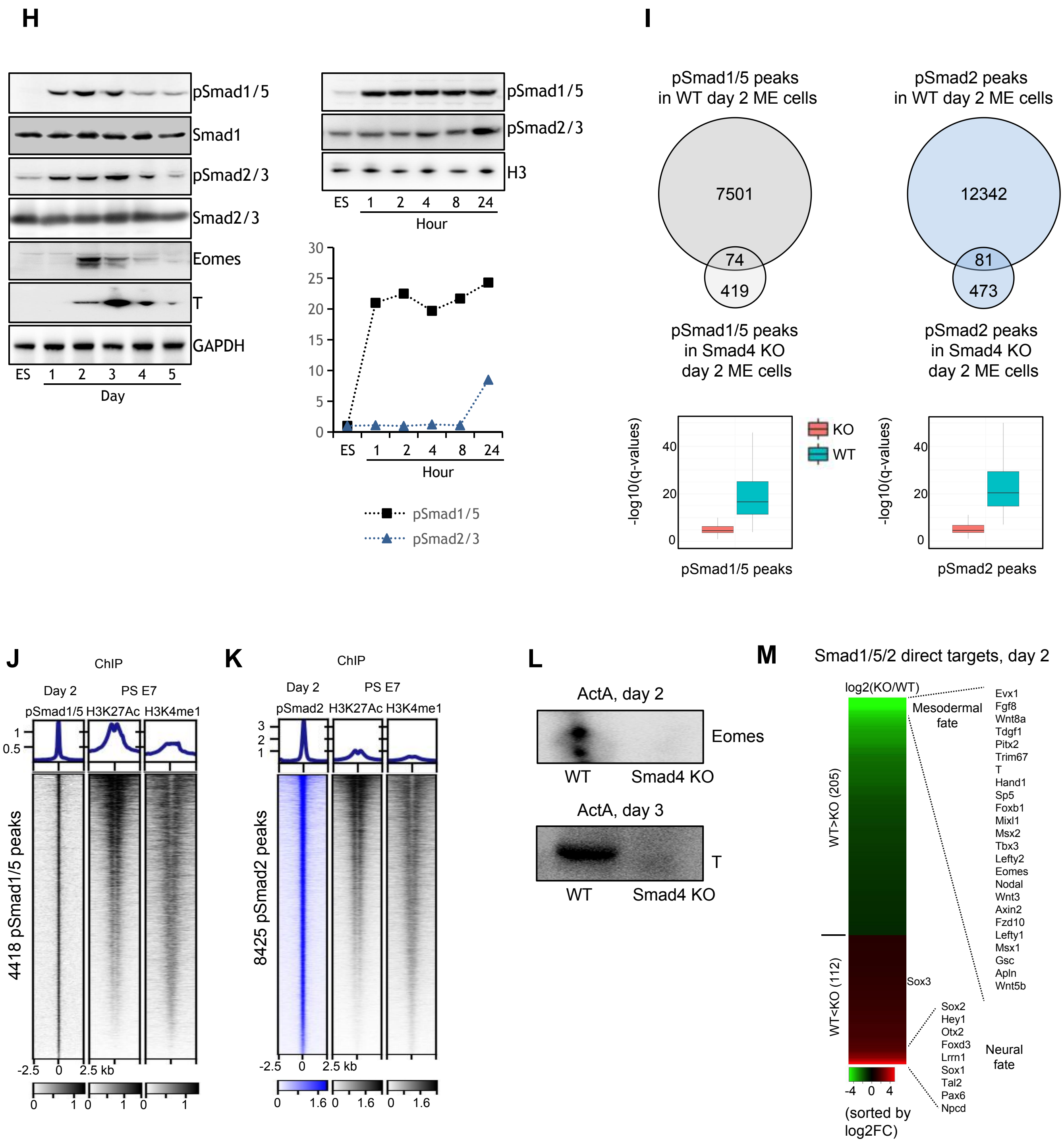

Development • Supplementary information

Fig. S1. Characterization of the *in vitro* differentiation system and DNA binding of Smads (related to Fig. 1)

(**A**) Differentiation efficiency assessed by FACS using ES cell line with T::H2B-mCherry reporter inserted into the translation start site of T located on a BAC. FACS dot plots indicating that over 90% of cells express T at differentiation day 3.
(**B**) Barplot showing fractions of cells expressing T::H2B-mCherry reporter during the differentiation time course assessed by FACS.
(**C-D**) Heatmap representation of the relative expression (z-score) of pluripotency factors, mesoderm progenitor and cardiac mesoderm marker genes (D), and EMT marker genes (E) throughout the course of differentiation. (RNA-seq, average of two replicates).
(**E**) Heatmap displaying the expression values (FPKM) of definitive endoderm and neuroectoderm marker genes throughout the course of differentiation. (RNA-seq, average of two replicates).
(**F**) Cardiomyocyte formation efficiency assessed by FACS using day 6 cells expressing cardiomyocyte marker Tnnt2 (510nm).
(**G**) Expression of cardiac markers Actc1 or Myl7 in differentiated WT aggregates at day 8 detected by immunofluorescence. Abbr.: Ctr, secondary antibody control.
(**H**) Time-course of Smad1/5 or Smad2/3 phosphorylation and Eomes or T expression detected by Western blot analysis. Quantification of the Smad1/5 and Smad2/3 phosphorylation levels relative to ES.
(**I**) Venn diagrams showing overlap of pSmad1/5 or pSmad2 peaks detected in WT and Smad4 KO day 2 ME cells. Peaks detected in Smad4 KO cells were excluded from further analyses. Boxplots showing distribution of the q-values for the corresponding peak sets.
(**J**) Heatmaps centered on pSmad1/5 peaks outside promoters, displaying ChIP-seq reads of pSmad1/5 in day 2 ME cells and H3K27Ac and H3K4me1 in E7 posterior primitive streak [35].
(**K**) Heatmaps centered on pSmad2 peaks outside promoters, displaying ChIP-seq reads of pSmad2 in day 2 ME cells and H3K27Ac and H3K4me1 in E7 posterior primitive streak [35].
(**L**) Expression of Eomes or T in WT or Smad4 KO cells treated with Activin A (ActA) for 2 or 3 days, respectively.
(**M**) Heatmap representation of the expression of pSmad1/5/2 direct target genes at day 2, sorted by log2 fold change (FC) of FPKM values in Smad4 KO or WT ME cells. The strongest activated and repressed target genes are listed on the right.

Development • Supplementary information

Figure S2

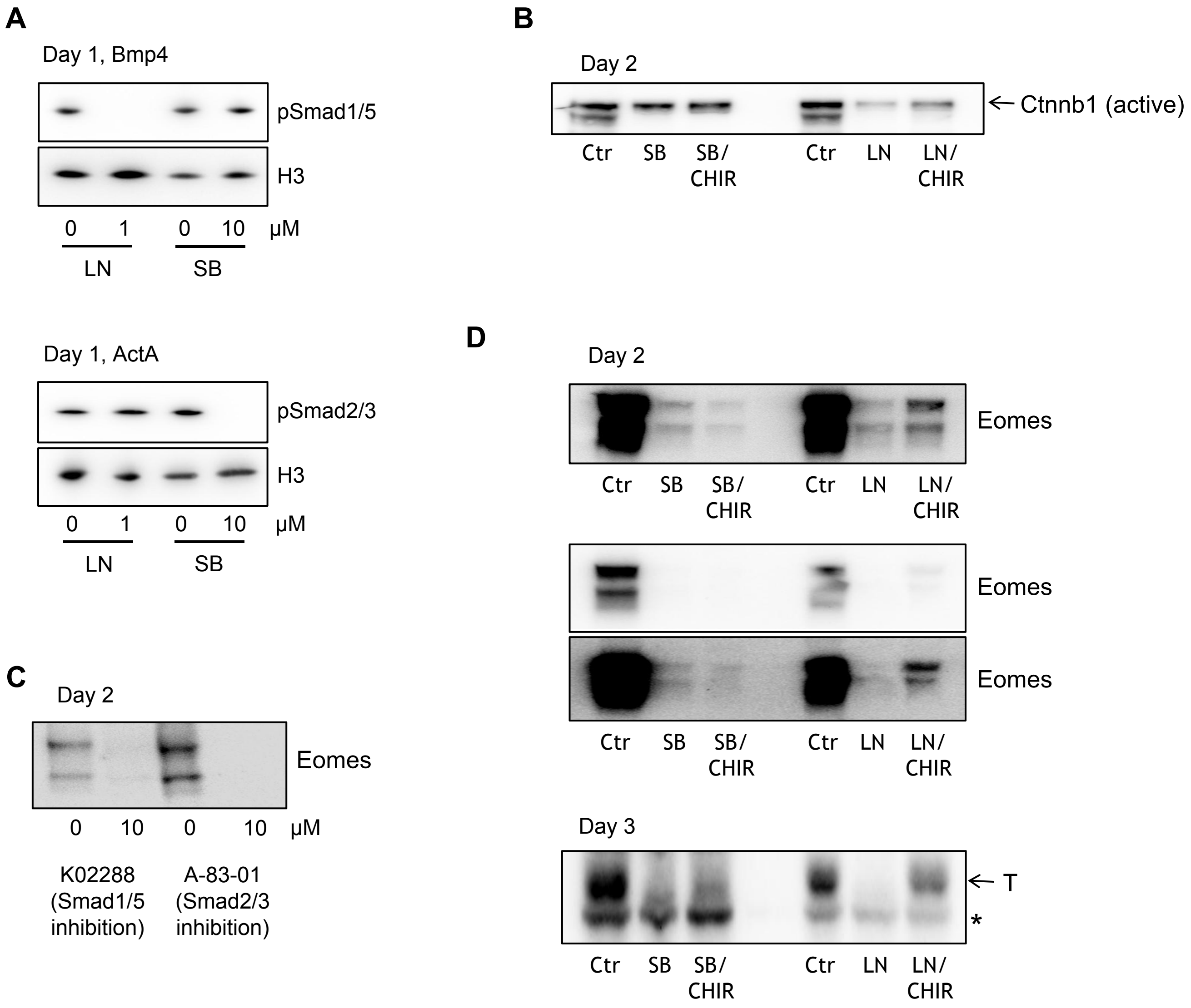
A
Day 1, Bmp4
pSmad1/5
H3
0 1 0 10 μM
LN SB
Day 1, ActA
pSmad2/3
H3
0 1 0 10 μM
LN SB
B
Day 2
Ctnnb1 (active)
Ctr SB SB/CHIR Ctr LN LN/CHIR
C
Day 2
Eomes
0 10 0 10 μM
K02288 (Smad1/5 inhibition)
A-83-01 (Smad2/3 inhibition)
D
Day 2
Eomes
Ctr SB SB/CHIR Ctr LN LN/CHIR
Eomes
Eomes
Ctr SB SB/CHIR Ctr LN LN/CHIR
Day 3
T
*
Ctr SB SB/CHIR Ctr LN LN/CHIR

Fig. S2. Formation of proper nascent and committed cardiac mesoderm requires simultaneous activity of pSmad1/5, pSmad2/3, and WNT signaling (related to Fig. 2)

(**A**) 1 µM LDN193189 (LN) or 10 µM SB431542 (SB) selectively inhibit pSmad1/5 or pSmad2/3 phosphorylation, respectively. pSmad1/5 and pSmad2/3 are detected by Western blot analysis. pSmad1/5 and pSmad2/3 phosphorylation was induced by Bmp4 or ActA, respectively.
(**B**) Nuclear accumulation of active Ctnnb1 is selectively blocked by 1 µM LDN (LN), as detected by Western blot analysis. Abbr.: Ctr, control.
(**C**) Eomes expression is repressed in day 2 ME cells by K02288 (inhibits Smad1/5 phosphorylation) as well as by A-83-01 (inhibits Smad2/3 phosphorylation).
(**D**) Western blot showing higher exposure of Fig. 2E, detecting Eomes expression (upper panel); Western blot analysis at low and high exposure showing Eomes protein expression in an independent experiment (middle panel). Independent experiment showing T expression by Western blot analysis (related to Fig. 2E; lower panel). Asterisk indicates a background band.

Figure S3

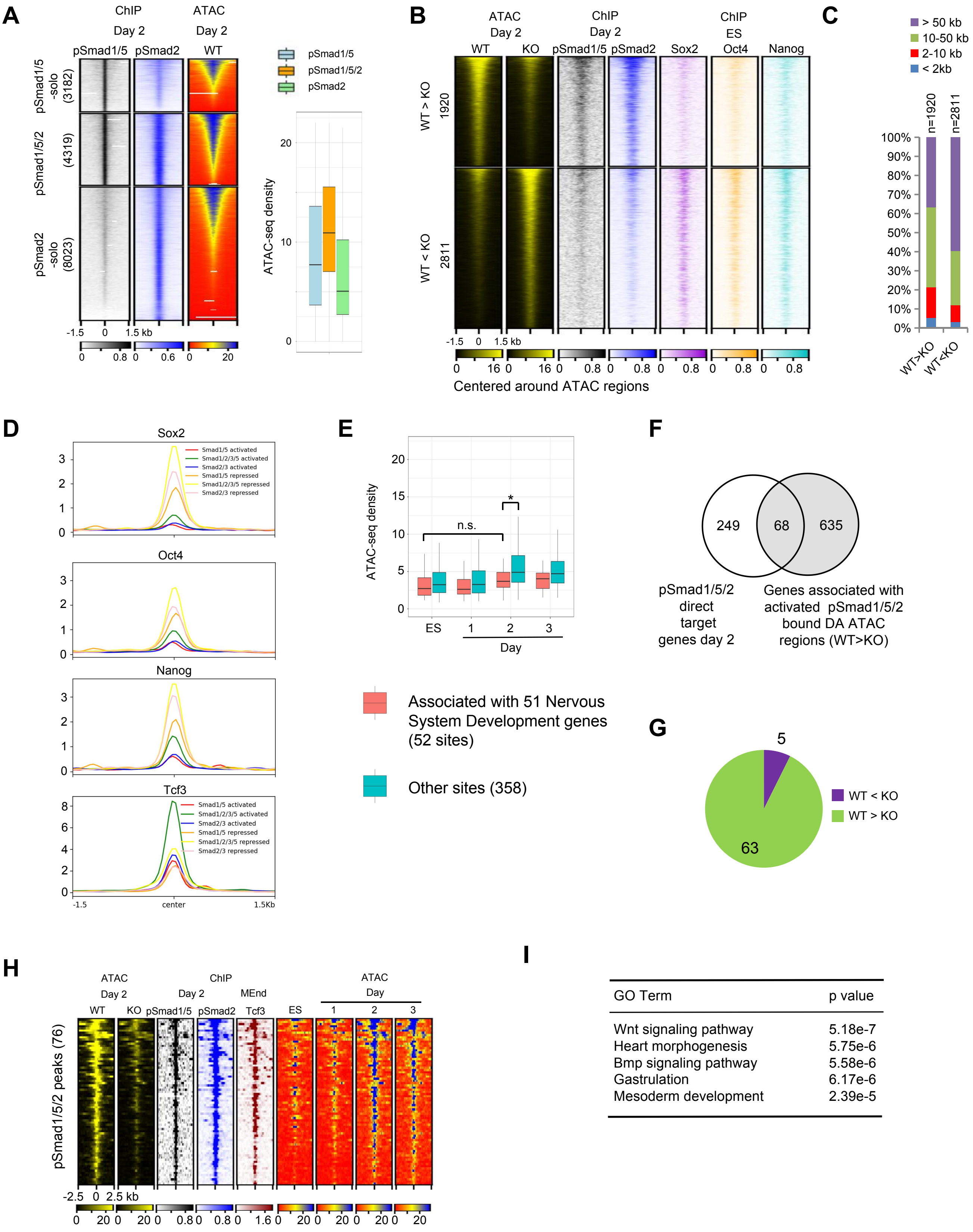

GO Term	p value
Wnt signaling pathway	5.18e-7
Heart morphogenesis	5.75e-6
Bmp signaling pathway	5.58e-6
Gastrulation	6.17e-6
Mesoderm development	2.39e-5

Development • Supplementary information

Fig. S3. Characterization of Smad-mediated chromatin regulation (related to Fig. 3)

(**A**) Heatmaps, clustered by pSmad1/5 and pSmad2 occupancy and centered on the peak summits, displaying ChIP-seq reads of pSmad1/5 or pSmad2 and ATAC-seq reads from the same sites in day 2 ME cells. Boxplot of the quantification of normalized ATAC-seq density is shown on the right.
(**B**) Clustered heatmaps displaying ATAC-seq reads in day 2 WT or Smad4 KO ME cells centered on sites activated or repressed by Smad activity, and the corresponding ChIP-seq reads of pSmad1/5, pSmad2, Sox2, Oct4 and Nanog.
(**C**) Barplot showing quantification of distances between DA regions in Smad4 KO relative to WT cells and nearest gene promoters for groups defined in (B).
(**D**) Average ChIP-seq density profiles of indicated TFs in clusters defined in Fig. 3C.
(**E**) Boxplot of the quantification of normalized ATAC-seq density during the differentiation course in sites associated with Nervous system Development GO term and other p-Smad2-solo bound sites defined in
(Fig. 3C, Fig. 3G). (Student's t-test, *$p < 5e-2$, n.s., not significant).
(**F**) Venn diagram showing overlap of pSmad1/5/2 direct target genes with genes bound by activated pSmad1/5/2-containing DA regions.
(**G**) Pie chart showing that the majority of pSmad1/5/2 direct targets associated with activated pSmad1/5/2-bound DA regions (see Fig. S3E) are Smad-activated (WT > Smad4 KO).
(**H**) Heatmaps centered on 76 pSmad1/5/2 peaks associated with 63 activated pSmad1/5/2 targets from (**F**), displaying ATAC-seq reads in WT or Smad4 KO day 2 ME cells, ChIP-seq reads of indicated TFs, and ATAC-seq reads during differentiation.
(**I**) GO terms enriched among the 63 activated pSmad1/5/2 target genes defined in (F).

Development • Supplementary information

Figure S4

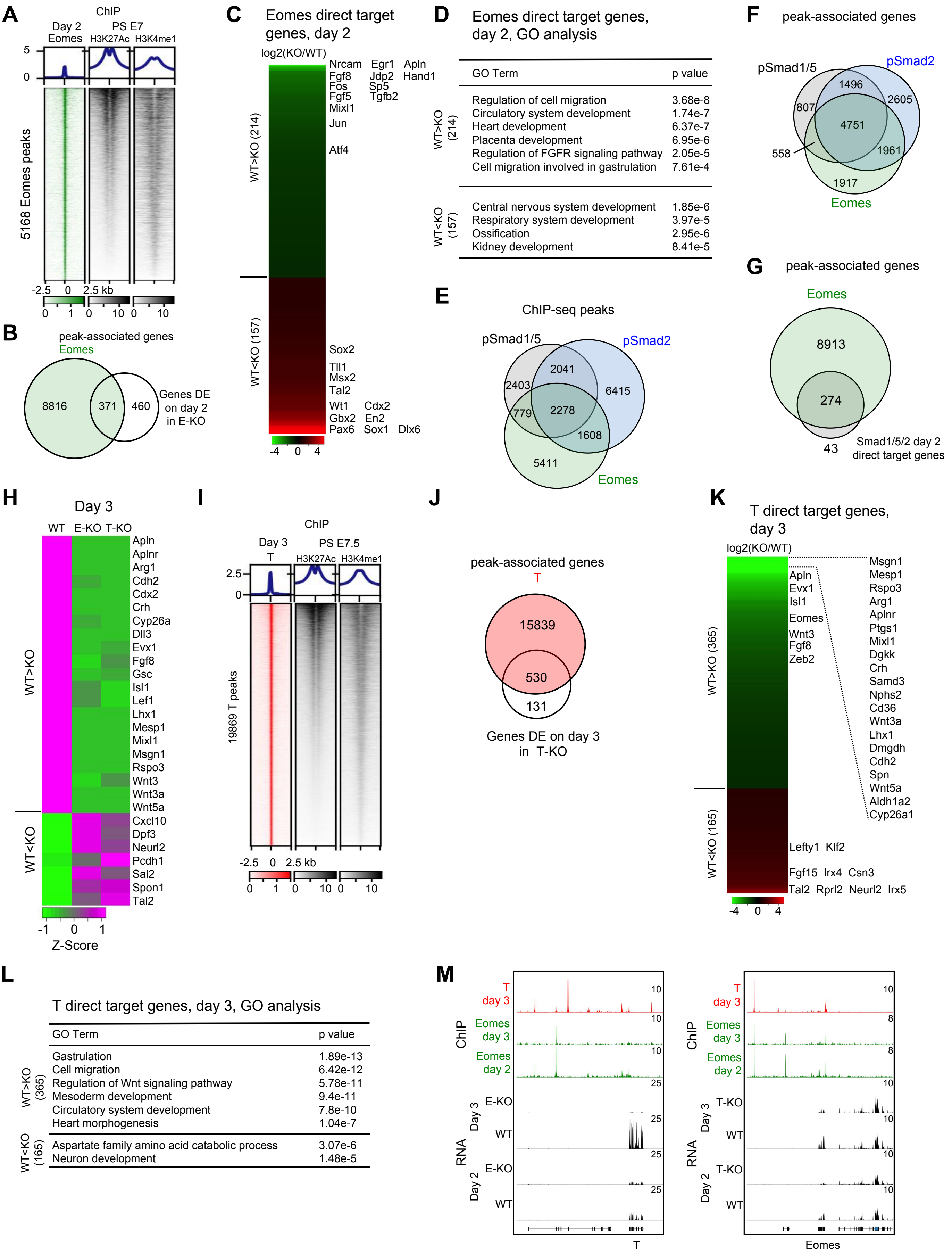

Development • Supplementary information

Fig. S4. DNA binding and target genes of Eomes and T (related to Fig. 5)

(**A**) Heatmaps centered on promoter-far Eomes peaks, displaying ChIP-seq reads of Eomes in day 2 ME cells, and H3K27Ac and H3K4me1 in E7 posterior primitive streak [35].

(**B**) Venn diagram showing overlap between genes bound by Eomes and genes DE between WT and Eomes KO cells. Genes in the intersection are putative direct targets of Eomes.

(**C**) Heatmap representation of Eomes day 2 direct target gene expression, sorted by log2 fold change (FC) of FPKM values in Eomes KO or WT ME cells. Selected activated and repressed target genes are listed on the right.

(**D**) GO terms enriched among activated (WT>KO) or repressed (WT<KO) Eomes direct target genes.

(**E**) Venn diagram showing overlap between pSmad1/5, pSmad2 and Eomes DNA binding sites in day 2 ME cells.

(**F**) Venn diagram showing overlap between genes bound by pSmad1/5, pSmad2, and Eomes in day 2 ME cells.

(**G**) Venn diagram showing overlap between genes bound by Eomes and direct target genes of
pSmad1/5/2 in day 2 ME cells.

(**H**) Heatmap representation of the expression of selected Eomes and T direct target genes in WT, Eomes KO (E-KO) or T KO (T-KO) day 3 ME cells. (RNA-seq, average of two replicates)

(**I**) Heatmaps centered on promoter-far T peaks, displaying ChIP-seq reads of T in day 3 ME cells, and H3K27Ac and H3K4me1 in E7.5 posterior primitive streak [35].

(**J**) Venn diagram showing overlap between genes bound by T and genes DE between WT and T-KO cells. Genes in the intersection are putative direct targets of T.

(**K**) Heatmap representation of the expression of T direct target genes at day 3, sorted by log2 fold change (FC) of FPKM values in T-KO relative to WT ME cells. Selected activated or repressed target genes are listed on the right.

(**L**) GO terms enriched among activated (WT>KO) or repressed (WT<KO) day 3 direct target genes of T.(**M**) T is a direct target of Eomes, and vice versa. Snapshots of ChIP-seq and RNA-seq tracks in WT, E-KO or T-KO ME cells at T and Eomes loci.

Development • Supplementary information

Figure S5

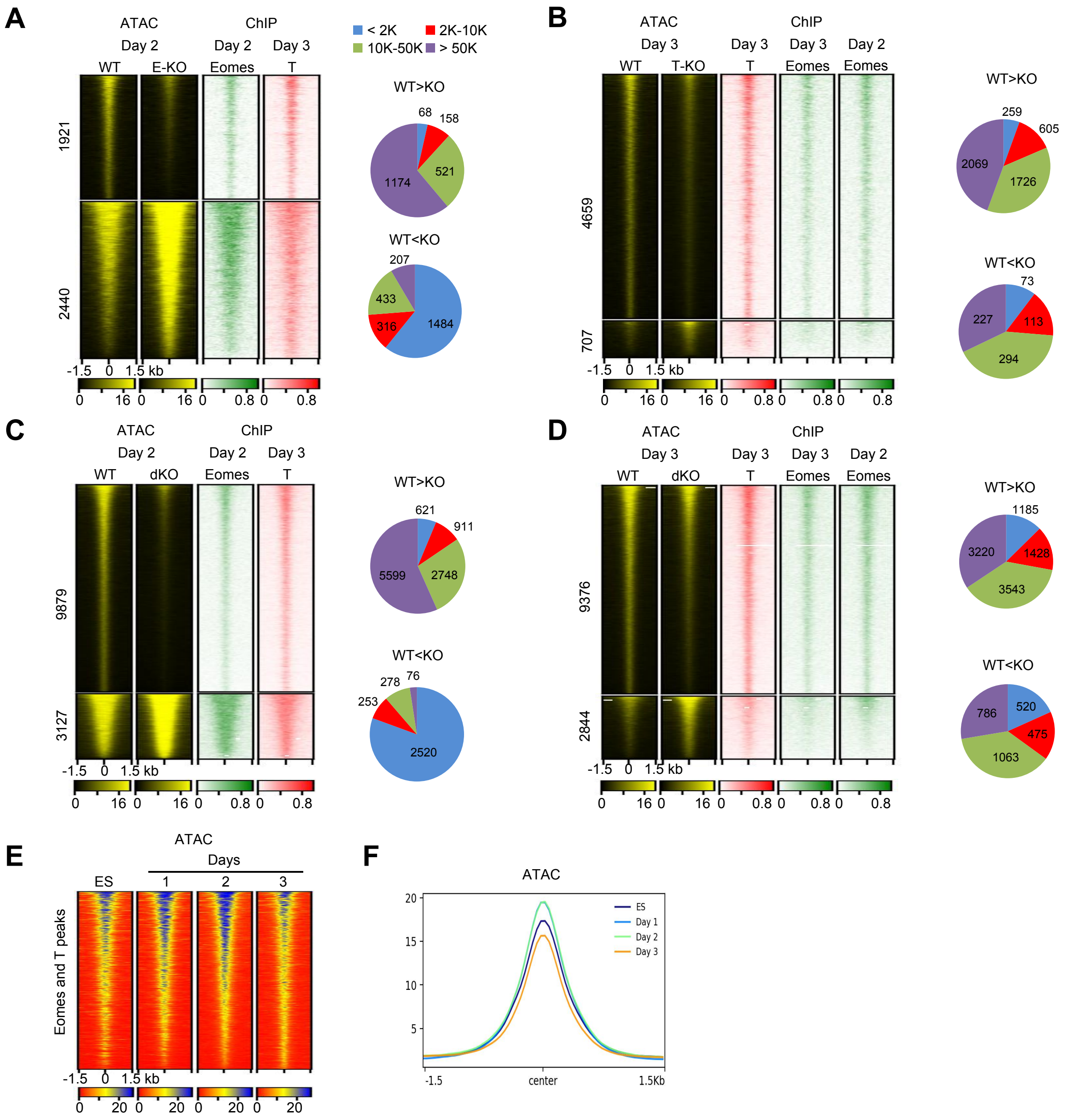
A
ATAC
ChIP
Day 2
Day 2
Day 3
WT
E-KO
Eomes
T
1921
2440
-1.5 0 1.5 kb
< 2K
2K-10K
10K-50K
> 50K
WT>KO
68
158
521
1174
WT<KO
207
433
316
1484
B
ATAC
ChIP
Day 3
Day 3
Day 3
Day 2
WT
T-KO
T
Eomes
Eomes
4659
707
WT>KO
259
605
2069
1726
WT<KO
73
227
113
294
C
dKO
9879
3127
621
911
5599
2748
278
76
253
2520
D
9376
2844
1185
3220
1428
3543
520
786
475
1063
E
ATAC
Days
ES
1
2
3
Eomes and T peaks
F
ATAC
ES
Day 1
Day 2
Day 3
center
1.5Kb

Fig. S5. Characterization of Eomes- and/or T-mediated chromatin regulation (related to Fig. 6)

(**A-D**) Clustered heatmaps displaying ATAC-seq reads in WT or (A) E-KO, (B) T-KO, (C,D) double KO
(dKO) ME cells, centered on activated or repressed DA sites, and the corresponding ChIP-seq reads of indicated TFs (left). Pie charts showing quantification of distances between the corresponding activated or repressed DA regions and nearest gene promoters (right).
(**E**) Heatmap displaying ATAC-seq reads centered on activated DA regions co-occupied by Eomes and T (see Fig. 6D).
(**F**) Average ATAC-seq density profiles in clusters from (E).

Development • Supplementary information

Table S1. GO term analysis for genes bound by pSmad1/5 and pSmad2 (related to Fig. 1).

GO Term	pSmad1/5 and pSmad2 common targets (6247)	pSmad1/5 only targets (1365)	pSmad2 only targets (4566)
Nervous system development	1.61e-27	2.84e-5	1.2e-5
Heart development	1.13e-18	ND	ND
Wnt signalling	4.4e-11	ND	ND
Mesoderm development	1.95e-8	ND	ND
Gastrulation	3.8e-8	ND	ND
TGFbeta signalling pathway	3.7e-7	ND	ND
Regulation of EMT	3.31e-5	ND	ND

Table S2. GO term analysis for direct target genes of pSmad1/5/2 (related to Fig. 1).

A pSmad1/5/2 direct targets, day 1

	GO Term	p value
WT>KO (110)	Mesoderm formation	1.91e-9
	BMP signaling pathway	1.63e-5
	Wnt signaling pathway	2.29e-5
WT<KO (89)	Nervous system development	4.21e-14
	Neuron differentiation	3.98e-9

B pSmad1/5/2 direct targets, day 2

	GO Term	p value
WT>KO (205)	Wnt signaling pathway	2.68e-11
	Heart development	3.17e-10
	Vasculature development	4.03e-8
	BMP signaling pathway	1.93e-7
	EMT	8.99e-7
	Mesoderm formation	4.87e-6
WT<KO (112)	Nervous system development	1.2e-11
	Neuron fate commitment	4.56e-5

Table S3. Lists of pSmad1/5, pSmad2, Eomes, and T direct target genes and genes associated with indicated sets of DNA loci (related to Figs. 1 - 6).

Click here to download Table S3

Table S4. Oligonucleotides used for generation and genotyping of knock-out cells. Primers used for RT-PCR.

Single stranded oligonucleotides for Eomes KO

Pair 1:	CACCGACATGGTCTACCCACGGCAT	F
	AAACATGCCGTGGGTAGACCATGTC	R
Pair 2:	CACCGTCCGGCGCCATAGAGCCACC	F
	AAACGGTGGCTCTATGGCGCCGGAC	R
Pair 3:	CACCGTCACGTAGATCTGCCCTCAA	F
	AAACTTGAGGGCAGATCTACGTGAC	R
Pair 4:	CACCGGTTTGCAGCAGGCGATTTG	F
	AAACCAAATCGCCTGCTGCAAACC	R

Eomes gene locus

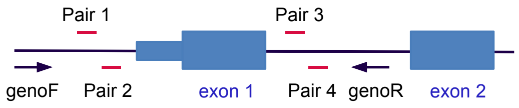

Eomes KO genotyping primers

genoF:	TGAAGGCCAGGAAGTCAGAAG	F
genoR:	GTCACCAGCTCAAACCGACTTC	R

Single stranded oligonucleotides for T KO

Pair 1:	CACCGCGCCACCCTCTCCACCTTCC	F
	AAACGGAAGGTGGAGAGGGTGGCGC	R
Pair 2:	CACCGTCGCCGGGCACAGAGAGCGC	F
	AAACGCGCTCTCTGTGCCCGGCGAC	R
Pair 3:	CACCGACGCACCTACCTGCCGTTCT	F
	AAACAGAACGGCAGGTAGGTGCGTC	R
Pair 4:	CACCGGCGAGTGCCCCTCGCCTCG	F
	AAACCGAGGCGAGGGGCACTCGCC	R

T gene locus

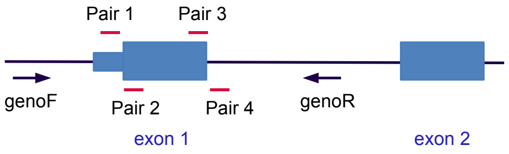

T KO genotyping primers

genoF:	GCAGCTGCTCGGTACTTCAAAG	F
genoR:	CAGCAGAGGGACAAATCTAGCCAAG	R

Primers used for qRT-PCR assays

CTTCTAATGGAGCCCCACCTG	F	Wnt3
GGGCCAGGGACCACCAAAT	R	
CTGGCGTACATGTTGAGCCT	F	Nodal
GGTCACGTCCACATCTTGCG	R	
GGGACAACTACGATTCATCCCA	F	Eomes
GGGCTTGAGGCAAAGTGTTG	R	
GGTATTCCCAATGGGGGTGG	F	T
TGTGTGGAGGGGAGAGAGAG	R	
GGATCCCCGCCTGCCTA	F	Mesp1
GCTGCTGAAGAGCGGAGAT	R	
CCCCGTCCATCAGAGAGCTA	F	Actc1
TGGGTTCTGTAGGCGTGCTA	R	
ACTGGGAAATGATGAGAATGGC	F	Pmm2
CCCCCAGATGCCCTTGAATA	R	

Table S5. RNA-seq data (FPKM values) of the RefSeq genes for the entire differentiation course and indication of genes associated with pSmad1/5, pSmad2, Eomes, and T ChIP-seq peaks; all direct target genes of pSmad1/5, pSmad2, Eomes, and T; and all genes associated with sites opened or closed in Smad4, Eomes and/or T KO cells compared to WT cells.

Click here to download Table S5

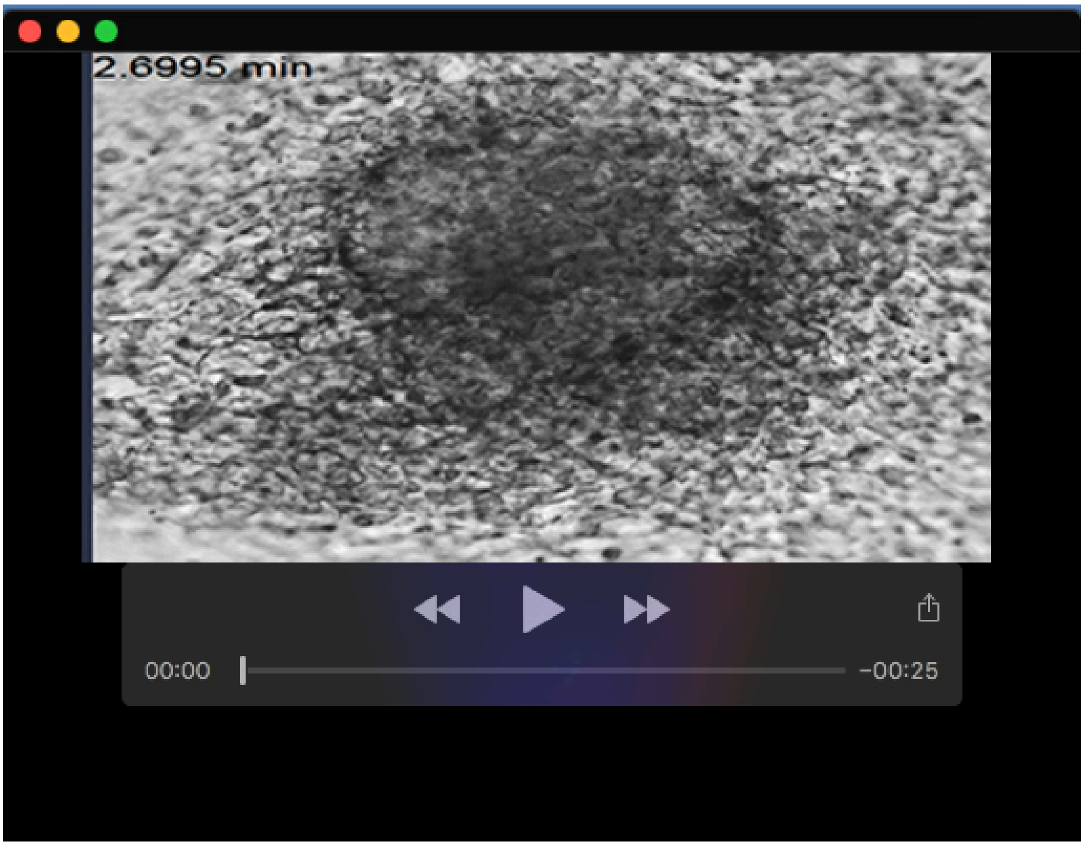

Movie 1. Capture of contracting cells on differentiation day 8.
